# Genetic architecture of acute hyperthermia resistance in juvenile rainbow trout (*Oncorhynchus* *mykiss*) and genetic correlations with production traits

**DOI:** 10.1186/s12711-023-00811-4

**Published:** 2023-06-12

**Authors:** Henri Lagarde, Delphine Lallias, Pierre Patrice, Audrey Dehaullon, Martin Prchal, Yoannah François, Jonathan D’Ambrosio, Emilien Segret, Ana Acin-Perez, Frederic Cachelou, Pierrick Haffray, Mathilde Dupont-Nivet, Florence Phocas

**Affiliations:** 1 Université Paris-Saclay, INRAE, AgroParisTech, GABI, 78350 Jouy-en-Josas, France; 2 SYSAAF, French Poultry, Aquaculture and Insect Breeders Association, 35042 Rennes, France; 3 Faculty of Fisheries and Protection of Waters, South Bohemian Research Center of Aquaculture and Biodiversity of Hydrocenoses, University of South Bohemia in České Budějovice, Zátiší 728/II, 389 25 Vodňany, Czech Republic; 4 Viviers de Sarrance, Pisciculture Labedan, 64490 Sarrance, France

## Abstract

**Background:**

Selective breeding is a promising solution to reduce the vulnerability of fish farms to heat waves, which are predicted to increase in intensity and frequency. However, limited information about the genetic architecture of acute hyperthermia resistance in fish is available. Two batches of sibs from a rainbow trout commercial line were produced: the first (N = 1382) was phenotyped for acute hyperthermia resistance at nine months of age and the second (N = 1506) was phenotyped for main production traits (growth, body length, muscle fat content and carcass yield) at 20 months of age. Fish were genotyped on a 57 K single nucleotide polymorphism (SNP) array and their genotypes were imputed to high-density based on the parent’s genotypes from a 665 K SNP array.

**Results:**

The heritability estimate of resistance to acute hyperthermia was 0.29 ± 0.05, confirming the potential of selective breeding for this trait. Since genetic correlations of acute hyperthermia resistance with the main production traits near harvest age were all close to zero, selecting for acute hyperthermia resistance should not impact the main production traits, and vice-versa. A genome-wide association study revealed that resistance to acute hyperthermia is a highly polygenic trait, with six quantitative trait loci (QTL) detected, but explaining less than 5% of the genetic variance. Two of these QTL, including the most significant one, may explain differences in acute hyperthermia resistance across INRAE isogenic lines of rainbow trout. Differences in mean acute hyperthermia resistance phenotypes between homozygotes at the most significant SNP was 69% of the phenotypic standard deviation, showing promising potential for marker-assisted selection. We identified 89 candidate genes within the QTL regions, among which the most convincing functional candidates are *dnajc7*, *hsp70b*, *nkiras2*, *cdk12*, *phb*, *fkbp10*, *ddx5*, *cygb1*, *enpp7*, *pdhx* and *acly.*

**Conclusions:**

This study provides valuable insight into the genetic architecture of acute hyperthermia resistance in juvenile rainbow trout. We show that the selection potential for this trait is substantial and selection for this trait should not be too detrimental to improvement of other traits of interest. Identified functional candidate genes provide new knowledge on the physiological mechanisms involved in acute hyperthermia resistance, such as protein chaperoning, oxidative stress response, homeostasis maintenance and cell survival.

**Supplementary Information:**

The online version contains supplementary material available at 10.1186/s12711-023-00811-4.

## Background

Aquaculture, which produced 7.5% of animal source proteins consumed worldwide in 2018, is a significant contributor to world food security [1]. The importance of aquaculture in global food production is expected to increase in the medium term as it grows faster than the other main food production sectors, with a mean annual growth of 4.6% between 2010 and 2020 [2]. However, this steady increase in aquaculture production over the past few decades could be jeopardised in the future by the effects of climate change. These effects are expected to be multiple, including an increase in chronic water temperature, more frequent and severe extreme weather events or changes in rainfall patterns, which will affect all levels of production by reducing growth and survival, occurrence of new and more frequent epidemics, or input shortages at fish farms [3].

As ectotherms, fish are particularly susceptible to changes in water temperature. Hence, the effects of climate change, both through increases in chronic temperature and acute hyperthermia conditions induced by more frequent and intense heat waves, are expected to substantially impact fish production [4]. Extreme temperature events are predicted to impact ectotherms populations more than climate warming [5]. Furthermore, the adaptative capacities of the European perch, *Perca* *fluviatilis*, were found to be greater for resistance to chronic hyperthermia stress compared to resistance to acute hyperthermia stress [6].

Hence, resilience of fish farms to heat wave events is a primary concern. Acute hyperthermia stress causes a series of physiological and behavioural changes in fish, starting with the release of catecholamines and cortisol, followed by an increase in plasma glucose and lactate, over-expression of genes associated with acute hyperthermia stress, such as heat shock proteins, and reduced or cessation of feeding [4]. The consequences of acute hyperthermia on production efficiency in fish farms are significant, among which growth losses and high mortality rates (e.g. [7]). Thus, adaptation strategies must be developed to guarantee food security by limiting production losses induced by heat waves. Promising strategies include engineering solutions, species diversification, improved farm management, nutrition, exercise training, and genetic improvement [8, 9]. Genetic improvement by selective breeding is an interesting solution to enhance fish resistance to acute hyperthermia conditions, as genetic gains are cumulative over generations and can be quickly disseminated to farms because of the high fecundity of fish.

Rainbow trout *Oncorhynchus* *mykiss* is the second most farmed salmonid species, with 959,600 tons produced worldwide in 2020 [2]. Like other salmonids, rainbow trout are particularly sensitive to acute hyperthermia stress, as their upper thermal resistance, the temperature at which they lose equilibrium, is usually between 26 °C and 30 °C [10, 11]. Breeding rainbow trout that are robust to acute hyperthermia stress would help fish farmers adapt to climate change.

Estimation of the heritability and its genetic correlations with production traits, and the identification of quantitative trait loci (QTL) for acute hyperthermia resistance are needed to optimise and assess the profitability of genetic improvement by selective breeding of this trait.

Heritability is a key parameter for estimating the expected genetic gain from selective breeding and thus for assessing the theoretical cost–benefit of selection on a trait [12]. Heritability of acute hyperthermia resistance was estimated to be 0.41 ± 0.07 in a North-American population of rainbow trout, which indicates that there is high potential for genetic improvement of this trait [13]. Nevertheless, the realised heritability was only 0.10 ± 0.05 after six generations of selection for acute hyperthermia resistance in the zebrafish *Danio* *rerio* [14]. This low realized heritability questions the relevance of genetics to improve fish resistance to acute hyperthermia stress.

Estimating genetic correlations between traits is also essential to evaluate the effect of selection for one trait on other traits of interest and vice-versa [12]. The three main traits of interest in rainbow trout breeding programs are growth, carcass yield, and fillet fat percentage at harvest age [15]. Estimating genetic correlations of these traits with acute hyperthermia resistance is all the more critical since trade-offs have been reported between growth and acute hyperthermia resistance in rainbow trout [11, 16]. The genetic correlation between acute hyperthermia resistance and growth was estimated to be − 0.03 ± 0.18 in a North-American commercial population of rainbow trout [13], but to date, this has not been studied in European commercial populations. Moreover, in the study of Perry et al. [13], fish were less than 1 year old, which is far from the harvest age required for rainbow trout filleting. To our knowledge, genetic correlations between acute hyperthermia resistance and carcass yield or fillet fat percentage have not been estimated in any fish species at any age.

The detection of QTL enables the identification of genetic markers that are associated with a phenotype of interest, which can then be used to improve the accuracy of selection by weighted genomic best linear unbiased prediction [17–20] or by marker-assisted selection, as has been shown to be successful for improving resistance to infectious pancreatic necrosis disease in Atlantic salmon *Salmo* *salar* [21–23]. Moreover, searching for candidate genes in QTL regions provides a better understanding of the mechanisms that are involved in acute hyperthermia resistance. Pioneering works studied the genetic architecture of acute hyperthermia resistance in the North-American population of rainbow trout used in Perry et al. [13] and identified several QTL but with large confidence intervals due to the low density of the marker array used [24–27]. More recent studies using high-density genotyping chips have shown that acute hyperthermia resistance is a polygenic trait and genes associated with this trait were identified in channel catfish *Ictalurus* *punctatus* and in large yellow croaker *Larimichthys* *crocea* [28, 29]. To date, such a study has not been conducted on rainbow trout.

The aim of the present study was to investigate the genetic architecture of acute hyperthermia resistance in a French commercial line of rainbow trout which has not previously been evaluated for this trait, by taking advantage of recent advances in genomics. The main objectives of our study were to (i) estimate genetic parameters for acute hyperthermia resistance in juveniles, (ii) estimate the genetic correlations of acute hyperthermia resistance with production traits at harvest age, (iii) detect QTL for acute hyperthermia resistance, and (iv) identify functional candidate genes for acute hyperthermia resistance.

## Methods

### Production of the fish

**Fig. 1:**
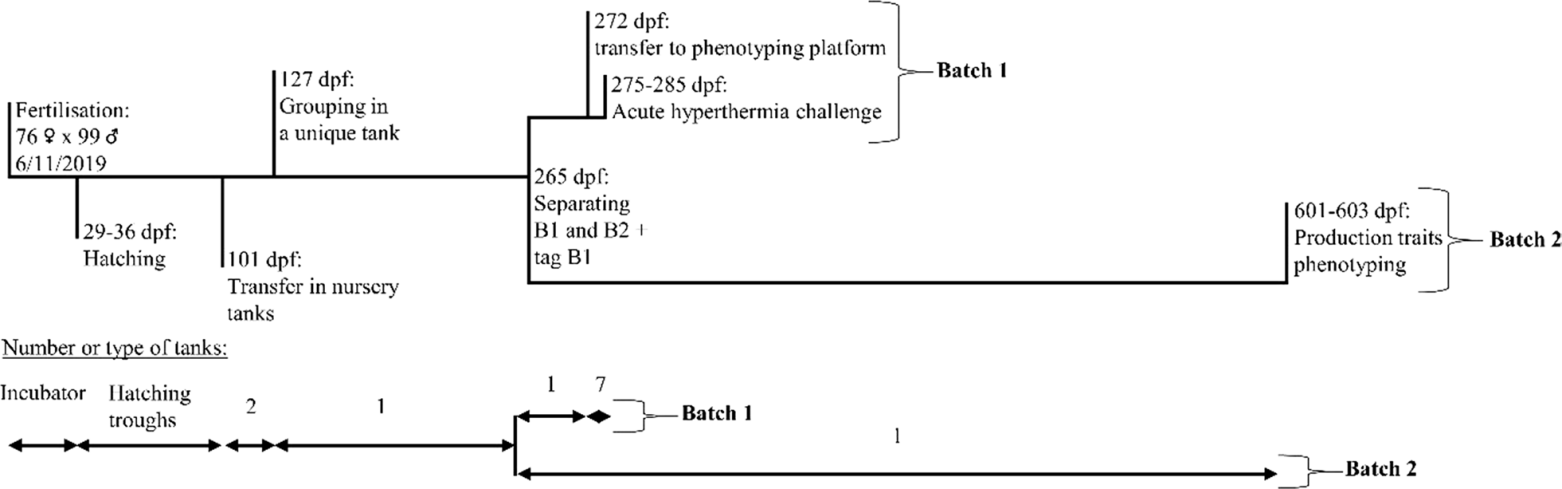
Batch 1 (B1, juveniles) and Batch 2 (B2, harvest size) rainbow trout production processes. dpf, day post-fertilisation

The process for producing the fish that were included in this study is summarised in Fig. 1. Fish were derived from a commercial line from the 'Viviers de Sarrance' (Sarrance, France) breeding company, that has been mass-selected during nine generations for growth, morphology for a ‘salmon-like’ shape, and gutted carcass yield measured by ultrasound tomography [30] and, more recently, for body colour and carcass yield based on a sib test. In November 2019, 76 two-year-old females were mated with 99 neomales (sex-reversed XX females used as sires) in 10 independent full-factorial blocks of 9 or 10 sires mated with 7 or 8 dams in the 'Labedan' hatchery (64490 Sarrance, France). Fin samples were collected from all parents and stored in 99% ethanol for later genotyping. The fertilised eggs were mixed and incubated in a 40-L cylindrical-conical incubator at 8.5 to 9 °C. At 29 days post-fertilisation (dpf), eyed-stage eggs were transferred to the 'Les Fontaines d'Escot' fish farm (64490 Sarrance, France). Eggs were randomly distributed in baskets that were placed in flow-through hatching troughs with circulating water at 13–14 °C, for hatching. Hatching occurred between 29 and 36 dpf. At 101 dpf, about 4500 fries were randomly transferred to two nursery tanks that were supplied with the same water source. At 127 dpf, fish were transferred to the ‘Viviers de Rébénacq’ fish farm (64260 Rébénacq, France), located 40 km from the previous farm. Fish from the two nursery tanks were grouped and reared in a 23-m^3^ concrete raceway. At 265-dpf, fish were randomly divided into two batches of approximately equal size named batch 1 (B1) and batch 2 (B2). Fish from batch B1 were tagged (Biolog-id, 1.4 × 8 mm) and fin-clipped for later DNA analysis. Then, B1 fish were placed in a second concrete raceway during 1 week before being transferred to the ANSES-SYSAAF Fortior Genetics platform (Plouzané, France) for phenotyping of acute hyperthermia resistance. Batch 2 (B2) included all remaining fish and was maintained in the 23-m^3^ concrete raceway. At 425 dpf, B2 fish (mean body weight of 300 g) were transferred to a 93-m^3^ concrete raceway, where they remained until phenotyping for production traits (between 600 and 604 dpf).

During growth, fish were fed ad libitum using extruded commercial feed (Le Gouessant, Lamballe, France). On the 'Viviers de Rébénacq' fish farm, fish were exposed to a minimum water temperature of 9.8 °C in February 2020 and a maximum of 14.5 °C in August 2020. The minimum oxygen concentration measured was 8.7 mg/L in August 2020. Density of the fish ranged from 6 to 24 kg/m^3^ in both batches. The survival rate of the B1 fish during the period from hatching till their transfer to the ANSES-SYSAAF Fortior Genetics platform was 65% and the survival rate of the B2 fish during the period from hatching till phenotyping was 63%.

### Phenotyping

#### Phenotyping for acute hyperthermia resistance (batch B1)

At 272 dpf, B1 fish (N = 1382) were transferred to the ANSES-SYSAAF Fortior Genetics platform for phenotyping of acute hyperthermia resistance. Transportation was done by truck under controlled conditions (transport duration: 10 h, fish density: 40 kg/m^3^, temperature: 12 ± 1 °C, O_2_ concentration: ~ 12.9 mg/L). When the fish reached the platform, they were randomly distributed in seven fibreglass 350-L tanks, with 197 ± 12 fish per tank for acclimation. Tanks were supplied with filtered and sterilised river water with a daily temperature range of 16–18 °C. Oxygen saturation level was maintained high by continuously and softly bubbling compressed air. The phenotyping was done by group, each fibreglass tank corresponding to one phenotyping group, at a rate of one group per day, covering a period from 275 to 285 dpf. Accordingly, the duration of acclimation, i.e. the time between the arrival at the phenotyping platform and the phenotyping day, ranged from 2 to 12 days, depending on when each group was phenotyped. The duration of acclimation will be discussed in detail later in the paper. Each group was starved 24 h before the phenotyping challenge.

**Fig. 2:**
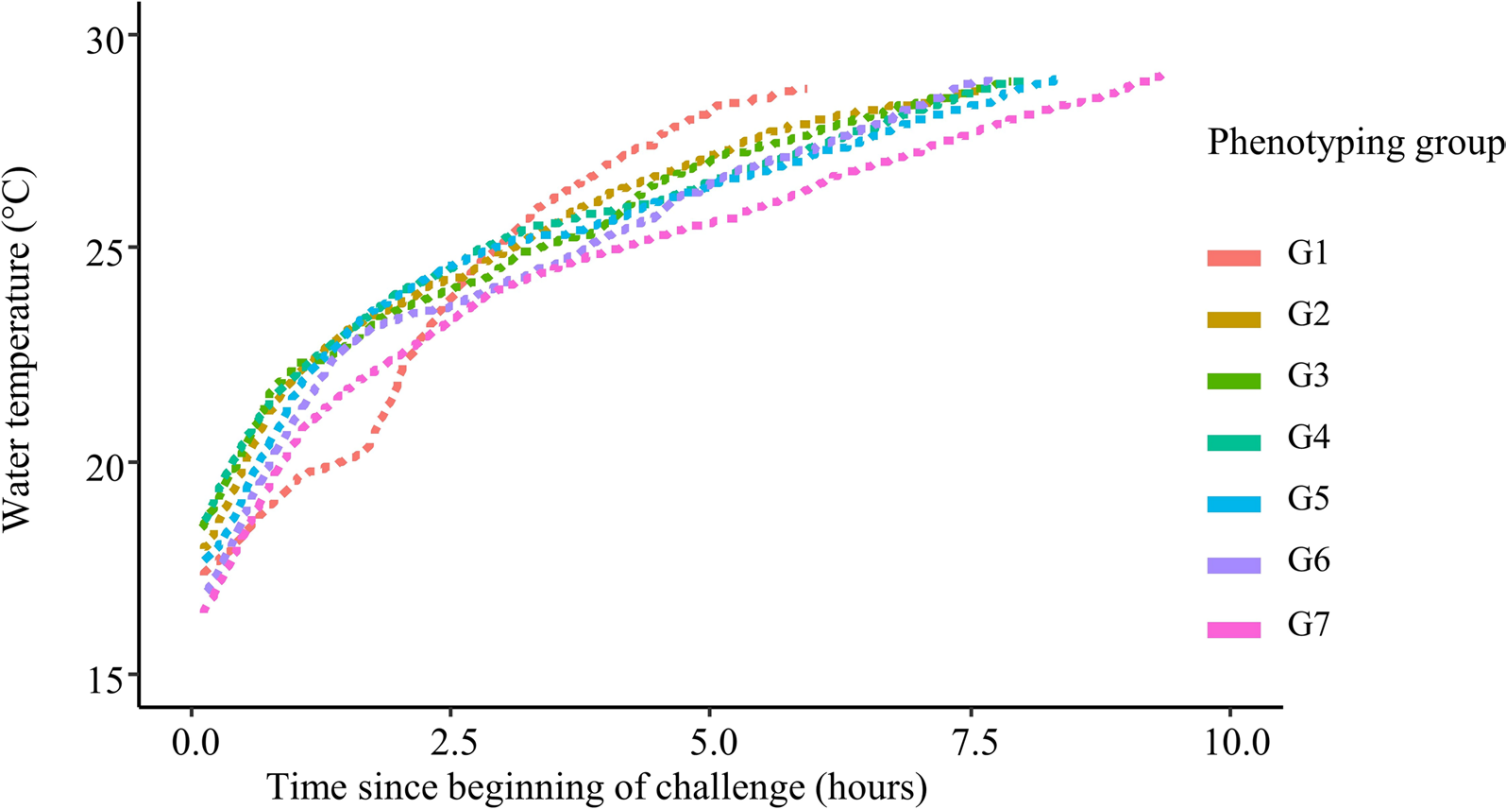
Kinetics of temperature increase in the seven rainbow trout groups of Batch 1 (B1, juveniles) of the acute hyperthermia challenge

Acute hyperthermia challenge was conducted as follows: at 9 am, fish from one group were transferred with a landing net to the challenge tank (350 L, fibreglass) that was supplied with the same river water as in the acclimation tanks. Once the transfer was completed, the temperature was gradually increased from the initial challenge tank water temperature (17.3 ± 0.7 °C, min: 16.1 °C in group 7 (G7), max: 18.4 °C in G4) at a rate of 3.1 °C/h during the first 1.5 h and then at a rate of 0.9 °C/h during the rest of the challenge by adding heated water from a buffer tank. The heating curves experienced by the seven groups are shown in Fig. 2. Oxygen saturation was maintained above 80% by softly bubbling pure O_2_. Above 27 °C, fish gradually began to lose equilibrium. Each fish losing equilibrium was removed from the tank and identified by its tag, the time was recorded and it was weighed and euthanised by an overdose of anaesthetic (Eugenol, 180 mg/L). Challenges ended when the last fish in the tank lost equilibrium. Temperature, O_2_ concentration and O_2_ saturation were recorded every 10 min using electronic probes (OxyGuard, Handy Polaris). ${\text{NH}}_{4}^{ + }$concentration, pH, and CO_2_ concentration were checked in the first three groups at the beginning and in the middle of the challenge, using commercial kits for ${\text{NH}}_{4}^{ + }$ (Tetra, Test), NH_3_/${\text{NH}}_{4}^{ + }$, and pH (JBL, pH test), and using a CO_2_ analyser (Oxyguard, CO_2_ Portable).

For batch B1, we recorded body weight (BW1) and acute hyperthermia resistance as the raw time to loss of equilibrium (rTLE). For reasons presented in the Results section, rTLE was centred to 0 and scaled to a standard deviation of 1 within each phenotyping group/day and will be referred to as TLE.

#### Phenotyping for production traits at commercial size (batch B2)

Fish from batch B2 (N = 1506) were phenotyped for production traits at commercial size over three days from 601 to 603 dpf. Each day, 502 ± 49 fish were slaughtered by bleeding in icy water immediately after netting. Fish were slaughtered in groups of 20 fish and stored on ice to avoid rigor mortis adverse effects. Once killed, the fish were phenotyped within 30 min maximum. First, a fin sample was collected on each fish for later genotyping and kept in 90% ethanol. The following phenotypes were then measured: body weight (BW2), fork length (FL), head weight (HeadW), headless gutted carcass weight (HGCW) as an indirect measurement of the fillet yield [31], viscera weight (ViscW), and headless gutted carcass yield (HGC%), calculated as the ratio of HGCW and BW2. Fillet fat percentage (Fat%) was estimated by micro-wave technology using a Distell Fish-Fatmeter^®^. The Fatmeter was applied at the anterior and posterior dorsal positions above the lateral line of the left side of the fish [32] and fat percentage was calculated as the mean of these two measurements. All weights were measured to the nearest 0.5 g, fork length to the nearest 0.5 mm, and total fat to the nearest 0.1%. When the difference between BW2 and the sum of HGCW, HeadW, and ViscW was larger than 10 g, the data for BW2 and HGCW were removed from the dataset (N = 24 individuals).

### Genotyping

The collected fin samples from the 99 sires, the 76 dams, the 1382 offspring of B1, and the 1506 offspring of B2 were genotyped on the INRAE genotyping platform Gentyane (Clermont-Ferrand, France). The 2888 offspring of B1 and B2 were genotyped for 57,501 SNPs (medium-density (MD) genotypes) with the 57K SNP AxiomTM Trout Genotyping array from Thermo Fisher [33]. The 175 parents were genotyped for 664,531 SNPs (high-density (HD) genotypes) with the 665K SNP AxiomTM Trout Genotyping array from Thermo Fisher [34]. For eight offspring from batch B2 and one sire, genotypes were obtained for less than 90% of the SNPs and were removed from the analysis. SNPs with probe polymorphisms and multiple locations on the Arlee strain rainbow trout genome assembly (accession number: GCA_013265735.3; [35]) were also discarded, following Bernard et al. [34]. In addition, only SNPs that had a call rate higher than 0.97, a minor allele frequency higher than 0.05, and a p-value > 0.00001 for the test for deviation from Hardy–Weinberg equilibrium were kept for further analysis. Finally, 30,325 SNPs with MD genotypes and 420,079 SNPs with HD genotypes remained after quality control.

Parentage assignment was done using the R package APIS [36], using 1000 SNPs from the MD chip that were equidistantly distributed across the genome with the positive assignment error rate set to 5%. All fish were assigned to their two parents, except for 55 fish in batch B1 and 48 in batch B2. Unassigned fish are probably mainly explained by missing genotypes for one sire. In batch B1, the mean number of phenotyped and genotyped progenies per sire, per dam, and per full-sib family were respectively 13.5 ± 5.1, 18.4 ± 6.5, and 2.4 ± 1.4 (unassigned individuals excluded). In batch B2, the mean number of phenotyped and genotyped progenies per sire, per dam, and per full-sib family were respectively 14.8 ± 5.3, 20.4 ± 8.5, and 2.5 ± 1.5 (unassigned individuals excluded).

As explained in the Results section, a maternal effect was found to be significant for the acute hyperthermia resistance phenotype and, thus, unassigned individuals were removed from the dataset, leaving 1327 fish for batch B1. Conversely, no significant maternal effect was found for the phenotypes measured in batch B2 and, thus, unassigned individuals were kept for this batch. The total number of analysed individuals in batch B2 was 1498 (1506 minus the 8 poorly genotyped individuals).

Based on the quality-filtered genotypes and pedigree information, the FImpute3 software [37] was used to impute missing genotypes for both the offspring and parents and to impute the offspring's MD genotypes to HD based on the parental reference HD genotypes.

### Data analysis

#### Estimation of genetic parameters

Genetic (co)variance components were estimated for all traits with the AIREML algorithm in the BLUPF90 software [38], using the following animal model:

1 $${y}_{ijk}=\mu +{day}_{i}+{dam}_{j}+{u}_{ijk}+{\varepsilon }_{ijk},$$

where ${y}_{ijk}$ is the performance of animal $k$, $\mu$ is the overall mean of the population, ${day}_{i}$ is the fixed effect of day of challenge $i$ (day/group of phenotyping for batch B1, day of slaughter for batch B2), ${dam}_{j}$ is the random effect of dam $j$, ${u}_{ijk}$ is the additive genetic effect of animal $k$, and ${\varepsilon }_{ijk}$ is the random residual error. A random dam effect was included only for TLE, as it was not significant for the other traits. Both pedigree and genomic relationship matrices were computed using the BLUPF90 software. The pedigree included 20,372 animals over 10 generations. The genomic matrix was built with imputed HD genotypes [39].

The heritability of each trait was estimated using univariate analysis with both the pedigree and genomic relationship matrices, as:

$${h}^{2}=\frac{{\sigma }_{u}^{2}}{{\sigma }_{p}^{2}},$$

where ${\sigma }_{u}^{2}$ is the estimate of the additive genetic variance and ${\sigma }_{p}^{2}$ is the estimate of the phenotypic variance.

The genetic correlation (${r}_{g}$) between two traits $x$ and $y$ was calculated using bivariate analyses with both the pedigree and genomic relationship matrices, as:

$${r}_{g}=\frac{cov({u}_{x}{u}_{y})}{\sqrt{({\sigma }_{{u}_{x}}^{2}{\sigma }_{{u}_{y}}^{2})}},$$

where $cov({u}_{x}{u}_{y})$ is the estimate of the additive genetic covariance between $x$ and $y$, and ${\sigma }_{{u}_{x}}^{2}$ and ${\sigma }_{{u}_{y}}^{2}$ are the estimates of the additive genetic variance of $x$ and $y$, respectively.

#### Genome-wide association study for acute hyperthermia resistance

A Bayesian variable selection model based on the Bayes Cπ approach implemented in the BESSiE software was used to localise QTL and estimate the proportions of genetic variance explained by the identified QTL [40, 41]. The effects of SNPs were estimated using the Markov chain Monte Carlo (MCMC) algorithm. At each cycle of the MCMC algorithm, only a fraction of the 420 K SNPs were assumed to have a non-zero effect on the phenotype and to follow a normal distribution N(0, ${\sigma }_{a}^{2}$), with ${\sigma }_{a}^{2}$ being the additive genetic variance.

The fitted model was:

2 $${TLE}_{ijk}=\mu +{day}_{i}+{dam}_{j}+\sum_{l=1}^{n}{\delta }_{lm}{{z}_{kl}a}_{l}+{\varepsilon }_{ijkm},$$

where ${TLE}_{ijk}$ is the acute hyperthermia resistance phenotype of animal $k$, $\mu$ is the overall mean of the population, ${day}_{i}$ is the fixed effect of day of challenge $i$, ${dam}_{j}$ is the random effect of dam $j$, $n$ is the total number of SNPs (420,079), ${\delta }_{lm}$ is an indicator variable: within a cycle $m$, ${\delta }_{lm}$= 1 if the effect of SNP $l$ is non-zero this cycle and ${\delta }_{lm}$= 0 otherwise, ${z}_{kl}$ is the genotype at locus $l$ for individual $k$ (coded as 0, 1, or 2), ${a}_{l}$ is the effect of the reference allele of SNP $l$, and ${\varepsilon }_{ijkm}$ is the residual effect. SNPs are assumed to have a mixture prior, among which most of them have a null effect with a probability (1 − π) and a small group of SNPs have a non-zero effect with probability π. The parameter π is unknown and, at each cycle, new values for π, and its counterpart 1 − π, are drawn from a beta distribution B(α, β) conditional on the number of SNPs included in the model in the previous sampling round, which corresponds to about α = 400 SNPs with a non-zero effect that are selected among β = 420,079 SNPs. The MCMC algorithm was run with 600,000 cycles and a burn-in period of 10,000 cycles. Results were saved every 40 cycles. Convergence was assessed using two approaches. First, plots of the posterior density of the sampled genetic and residual variances were verified by visual inspection. Second, genomic breeding values estimated from two differently seeded runs of the MCMC algorithm were highly correlated (r > 0.99).

The Bayes factor (BF) was used to quantify the degree of association between a SNP and resistance to acute hyperthermia. For the $i$th SNP, the BF is equal to $\frac{{P}_{i}/(1-{P}_{i}) }{\pi /(1-\pi )}$, where ${P}_{i}$ is the probability of SNP $i$ to be included in the model with a non-zero effect. As proposed by Kass et al. [42], a logBF value (equal to twice the natural logarithm of BF) greater than or equal to a threshold of 6 at the most strongly associated SNP (hereafter referred to as peak SNP) was used as strong evidence for the presence of a QTL.

Following Michenet et al. [43], a credibility interval was computed around the peak SNP for each QTL that included all SNPs for which logBF ≥ 3 in a sliding window of 200 kb on both sides of the peak SNP. Genes within QTL credibility intervals were annotated with the NCBI *O.* *mykiss* Arlee genome assembly (GCA_013265735.3 USDA_OmykA_1.1; [35]).

#### Time to loss of equilibrium depending on genotypes at SNP peaks

We analysed the acute hyperthermia resistance phenotype of individuals according to their genotype at the peak SNP of the detected QTL. Significance of the difference in TLE, corrected for the dam and day effects, between the two homozygous genotypes at each peak SNP was analysed by Anova Tukey tests. The difference was expressed as a % of the phenotypic standard deviation. The dominance effect was quantified at each SNP peak as the difference between the mean TLE of the heterozygous genotype and the average of the means of the two homozygous genotypes and its significance was tested using a one sample t-test, using a p-value threshold of 0.05.

#### Validation of identified QTL in isogenic lines of rainbow trout

In a previous experiment, we had measured the acute hyperthermia resistance phenotype of six isogenic lines of rainbow trout at 185 dpf (6 months) and at 457 dpf (15 months), using a protocol similar to that used in the present study [16]. Isogenic lines are powerful experimental genetic resources, because within each isogenic line, fish share the same genotype while the different lines represent a sample of the genetic variability of the INRAE synthetic population from which they were derived [44].

At 185 and 457 dpf, the most resistant isogenic line was A32h and the most sensitive was A22h [16]. Consequently, in this paper, we refer to A32h as the resistant isogenic line and A22h as the sensitive isogenic line. The four other isogenic lines were phenotyped as described in Lagarde et al. [16] and had either an intermediate ranking or their ranking in terms of resistance changed between 185 and 457 dpf and were, therefore, not considered here.

Isogenic lines A32h and A22h are heterozygous lines since they were produced by mating females from a unique homozygous isogenic line named B57 with males from the homozygous isogenic lines A32 and A22 and, thus they share the same maternal genetic background but have different paternal genetic contributions. These two lines were previously sequenced to establish a catalogue of variants [34]. In this paper, since the B57 maternal genetic sequence was identical between the two lines, we considered only the A22 and A32 paternal genetic sequences and we checked if some of the genetic polymorphisms associated with acute hyperthermia resistance detected in commercial populations were shared with those of the isogenic lines. Thus, among the SNPs identified to be strongly associated with TLE in the present study for the commercial population, i.e. which had a logBF > 6, we searched for those that were present in the genomes of A22 and A32. For the SNPs that had a logBF > 6, we examined whether the resistant A32h isogenic line carried the favourable allele (for a given SNP $l$, reference allele if ${a}_{l}$ > 0 or the other allele if ${a}_{l}$ < 0), as estimated in the GWAS on the commercial population.

## Results

### Descriptive statistics for the recorded phenotypes

In the present study, two batches of all-female rainbow trout originating from the same families were phenotyped at 275–285 dpf for acute hyperthermia resistance (B1) or 600–604 dpf for production traits (B2). Descriptive statistics for the recorded phenotypes are in Table 1.

**Table 1:** Descriptive statistics for acute hyperthermia resistance, growth, carcass yield and fatness in batches B1 and B2

Batch	Name	Trait	N	Mean	SD	Min.	Max.	CV (%)
B1	rTLE	Raw resistance to acute hyperthermia (min)	1327	412.2	56.1	261.1	564.4	13.6
	TLE	Standardised resistance to acute hyperthermia (no unit)	1327	0.0	1.0	− 4.5	2.7	NA
	BW1	Body weight of batch B1 (g)	1327	87.4	12.2	51.5	139.1	13.9
B2	BW2	Body weight of batch B2 (g)	1470	977.5	106.2	491	1483	10.9
	FL	Fork length (mm)	1497	446.5	15.2	375	491	3.4
	Fat%	Total fat content in muscle (%)	1497	4.9	1.2	1.3	9.6	24.3
	HGC%	Headless gutted carcass yield (%)	1469	78.7	1.0	74.2	81.7	1.3

*N* number of fish after filtering out poor-quality phenotypes and genotypes, *SD* standard deviation, *Min*. minimum value, *Max*. maximum value, *CV* coefficient of variation (SD/mean × 100), *NA* not applicable

**Fig. 3:**
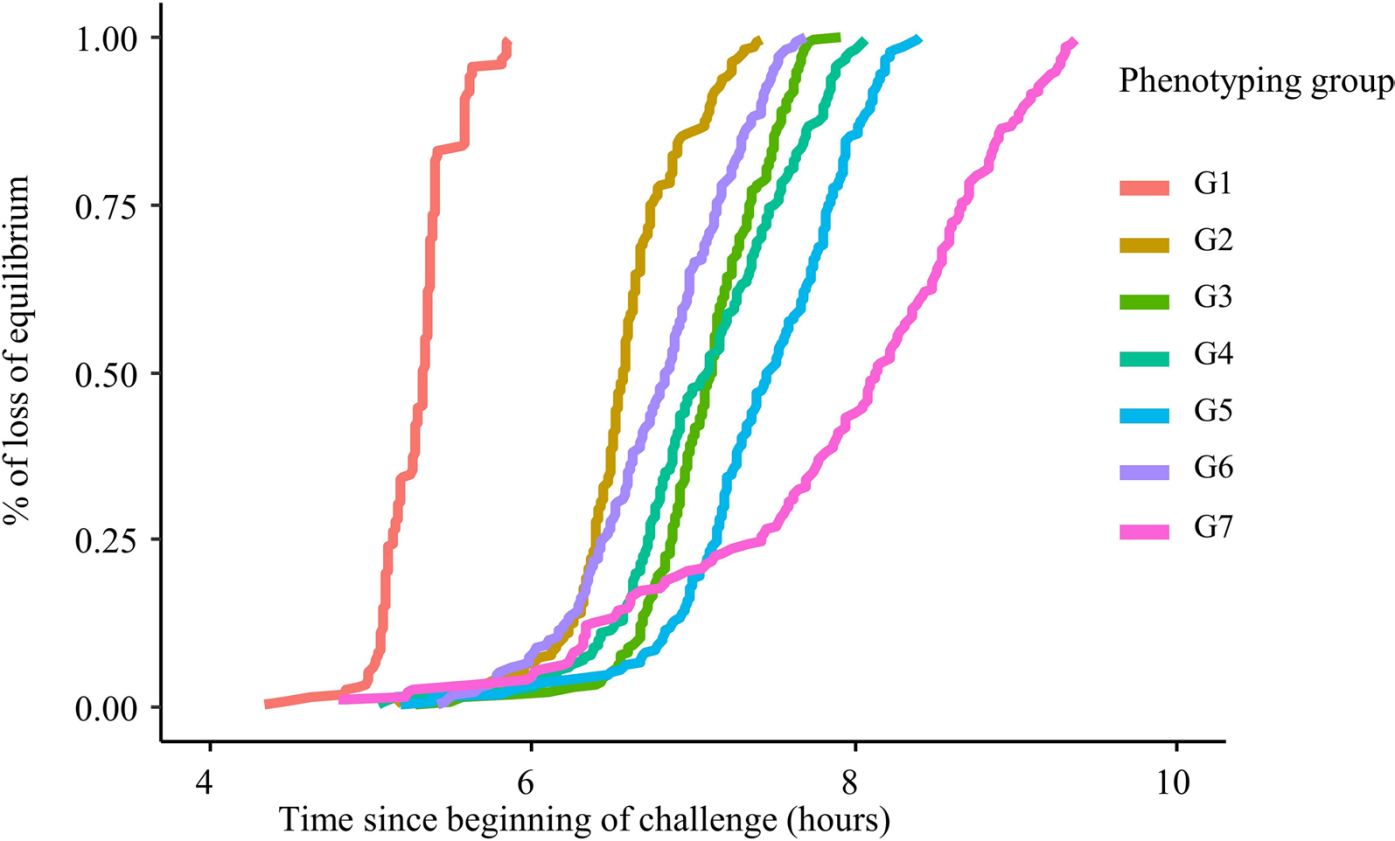
Kinetics of cumulative loss of equilibrium in the seven rainbow trout groups of Batch 1 (B1, juveniles) of the acute hyperthermia challenge

Average BW1 in batch B1 was 85.6 ± 12.0 g. The mean rTLE was 412 ± 56 min across all phenotyping groups but there were significant between-groups differences for rTLE (ANOVA, N = 1327, df = 6; F value = 402.7; P < 2.2e−16) and in the standard deviation of rTLE (Levene's statistic, N = 1327, df = 6; F value = 56.9; P < 0.001). As shown in Table 2, fish in phenotyping group G1 lost equilibrium quickly with a mean of 319 min and a small SD of ± 14 min, fish in G2, G3, G4, G5, and G6 showed intermediate and similar rTLE means (from 395 to 447 min) and SD (from 23 to 34 min), and fish in G7 lost equilibrium the latest with a mean rTLE of 474 min and a large SD of ± 62 min. The kinetics of loss of equilibrium are shown in Fig. 3. To correct for these between-group differences, rTLE was centred to 0 and scaled to a standard deviation of 1 within each phenotyping day to obtain a corrected phenotype, TLE. Minimum and maximum values for TLE across groups were − 4.5 and 2.7, respectively (Table 1).

**Table 2:** Mean and standard deviation of raw TLE (rTLE) in the acute hyperthermia phenotyping groups in batch B1

Phenotyping group	rTLE mean	rTLE SD	N
G1	318.7	14.1	187 (194)
G2	394.9	23.2	196 (202)
G3	424.8	24.4	192 (204)
G4	424.9	34.2	195 (200)
G5	446.8	34.0	199 (207)
G6	407.4	30.3	196 (202)
G7	473.8	61.6	162 (173)

The number of acclimation days, i.e. from arrival of fish on the phenotyping platform to when each group was phenotyped, ranged from 2 days for G1 to 12 days for G7. The number of days of acclimation and the temperature in the challenge tank at 6 h were strongly correlated with the between-group differences in mean and SD of rTLE. Correlations of the number of acclimation days for each phenotyping group with the mean and SD of rTLE within each group were 0.97 and 0.89, respectively. The temperature in the challenge tank 6 h after the start of the challenge was also strongly correlated with the group mean and SD of TLE, with correlation coefficients of − 0.94 and − 0.94, respectively. The number of acclimation days and the temperature in the challenge tank 6 h after the start of the challenge were also highly correlated together with a coefficient of correlation of − 0.93: phenotyping groups with the largest number of acclimation days were also those for which the temperature in the challenge tank at 6 h was lowest.

The ${\text{NH}}_{4}^{ + }$ concentration and the pH were measured at precise time points for the three first groups. The ${\text{NH}}_{4}^{ + }$ concentration was below the colorimetric detection threshold at the start of each challenge for groups that had a pH of 7.4, while it ranged from 0.25 to 1.5 mg/L for the three groups with a pH of 7.2 ± 0.3, 3 h after the start of the challenges, and from 2 to 3 mg/L for the three groups with a pH of 7.2 ± 0.4, 6 h after the start of the challenges. The CO_2_ concentration was measured for the first two groups at the start of the challenge (9 mg/L) and 3 h thereafter (12 mg/L).

The descriptive statistics for the production phenotypes at commercial size (B2) are also in Table 1.

Phenotyping groups are the seven acute hyperthermia resistance phenotyping groups from G1 (group/day 1) to G7 (group/day 7); rTLE mean, mean of raw resistance to acute hyperthermia (in min); rTLE SD, standard deviation of raw resistance to acute hyperthermia; N, number of fish phenotyped, genotyped and assigned. Between brackets, number of fish phenotyped.

### Genetic parameters for TLE and the production traits

Estimates of heritability and of genetic correlations based on pedigree (BLUP, see Additional file 1: Table S1) or genomic information (GBLUP, Table 3) gave very similar results for all traits. The GBLUP estimates had a lower SE than the BLUP estimates (Table 3) and (see Additional file 1: Table S1). Hence, unless specified, in the rest of the paper, the results shown and discussed are those from the GBLUP model.

**Table 3:** Estimates of genetic parameters based on the GBLUP model between traits measured in batches B1 (juveniles) and B2 (harvest size)

	TLE	BW1	BW2	FL	Fat	HGC%
TLE	**0.29 ± 0.05**	− 0.49 ± 0.13	0.00 ± 0.12	− 0.02 ± 0.12	0.13 ± 0.10	0.11 ± 0.10
BW1	− 0.07 ± 0.03	**0.19 ± 0.04**	0.45 ± 0.13	0.35 ± 0.13	− 0.05 ± 0.12	− 0.20 ± 0.11
BW2	NA	NA	**0.26 ± 0.04**	0.75 ± 0.05	− 0.15 ± 0.10	0.08 ± 0.10
FL	NA	NA	0.86 ± 0.01	**0.31 ± 0.04**	− 0.25 ± 0.09	0.02 ± 0.09
Fat	NA	NA	0.10 ± 0.03	0.01 ± 0.03	**0.45 ± 0.04**	0.28 ± 0.08
HGC%	NA	NA	0.13 ± 0.03	0.14 ± 0.03	0.19 ± 0.03	**0.61 ± 0.04**

Heritability estimates are in bold on the diagonal, genetic correlations are in the upper triangle, phenotypic correlations are in the lower triangle for standardised time to loss of equilibrium in the acute hyperthermia challenge (TLE); *BW1* body weight of batch 1, *BW2* body weight of batch 2, *FL* fork length, *Fat%* fillet fat percentage, *HGC%* headed gutted carcass yield. All values are given ± standard error. *NA* not applicable as traits were measured on different individuals

The BLUP and GBLUP models were tested with and without a random dam effect for all traits. Including a random dam effect improved the Akaike information criterion (AIC) of the BLUP and GBLUP models for TLE (for BLUP: AIC was equal to 3804 and 3809, with and without a dam random effect, respectively), but not for the other traits (data not shown), thus this effect was only kept in models for TLE.

The maternal effect was found to explain 7.0 ± 3.0% of the phenotypic variance for TLE in the BLUP and 6.0 ± 3.0% in the GBLUP model. Estimates of pedigree-based and of genomic heritability of TLE were similar, i.e.0.24 ± 0.07 and 0.29 ± 0.05, respectively. The two groups with extreme means and SD for TLE, G1 and G7, had limited impact on the estimate of heritability of TLE, as the estimates of pedigree-based and genomic heritability were 0.31 ± 0.05 and 0.28 ± 0.05, respectively, when the G1 and G7 data were removed. As a result, the G1 and G7 data were kept in the dataset. Estimates of heritability were moderate for BW1 (0.19 ± 0.04), BW2 (0.26 ± 0.04), and FL (0.31 ± 0.04), and high for Fat% (0.45 ± 0.04) and HGC% (0.61 ± 0.04). As shown in Table 3, the estimate of the genetic correlation of TLE with BW1 was clearly negative (− 0.49 ± 0.13), while estimates of TLE with BW2 and other production traits (FL, Fat%, and HGC%) were all close to zero (between − 0.02 ± 0.12 and 0.13 ± 0.10). Phenotypic correlations could only be estimated between traits that were measured within the same batch (see lower triangle of Table 3). The phenotypic correlation of TLE with BW1 was negative but close to 0: − 0.07 ± 0.03.

### Genome-wide association study for acute hyperthermia resistance

**Fig. 4:**
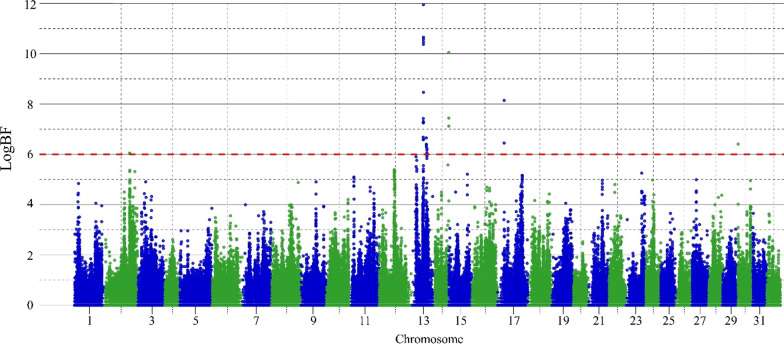
Manhattan plot of QTL detected for acute hyperthermia resistance trait in rainbow trout in Batch 1 (B1, juveniles). The red horizontal line corresponds to the QTL evidence threshold (logBF ≥ 6)

A GWAS was performed for TLE using HD genotypes (see Fig. 4 for the Manhattan plot). Seven QTL were detected with a logBF ≥ 6 for the peak SNP and these were located on five chromosomes. Among these, the QTL detected on chromosome 30 was discarded, as no other SNP had a logBF ≥ 3 in a sliding window of 200 kb around the peak SNP (Affx-1237752048). Visual inspection of the genotyping clusters indicated that the genotyping of this SNP was of poor quality and that the high logBF value should be considered an artefact. Therefore, we considered only the six other QTL, which will be referred to as TLE2-1, TLE13-1, TLE13-2, TLE13-3, TLE14-1, and TLE17-1 (Table 4).

**Table 4:** Effect and location of the QTL detected for acute hyperthermia resistance

QTL name	Chr	Peak SNP identifier	Peak SNP position (Mb)	LogBF	% variance explained by peak SNP	MAF	QTL region (Mb)	N of SNPs in QTL	% variance explained by QTL	N of genes in QTL region
TLE2-1	2	Affx-1237558532	78.97	6.05	0.03	0.34	[78.62–79.45]	160	0.63	8
TLE13-1	13	Affx-1237409861	36.68	11.95	1.13	0.42	[35.86–36.93]	317	4.60	35
TLE13-2	13	Affx-1237407727	45.63	6.66	0.04	0.46	[45.61–45.76]	66	0.20	9
TLE13-3	13	Affx-1237411204	47.37	6.24	0.02	0.24	[47.38–48.02]	205	0.40	25
TLE14-1	14	Affx-1237353641	42.93	10.05	0.25	0.31	[42.94–43.08]	15	0.37	4
TLE17-1	17	Affx-1248428573	20.73	8.15	0.07	0.21	[20.61–20.73]	13	0.11	1

Chr. Chromosome, Mb stands for mega-base-pair; % variance explained by peak SNP, % of genetic variance explained by the peak SNP; MAF, minor allele frequency; % variance explained by QTL, % of genetic variance explained by all the SNP included in the QTL region; N, number

The peak SNP for TLE13-1 showed strong evidence for a QTL, with a logBF above 10. The whole QTL TLE13-1 explained more than 4% of the genetic variance of TLE (Table 4). The five other QTL each explained less than 1% of the genetic variance (between 0.11% and 0.63%). This suggests that resistance to acute hyperthermia is a highly polygenic trait.

The size of the credibility intervals of QTL ranged from 0.12 to 1.07 Mb (Table 4). Eighty-nine distinct genes were identified across the six QTL regions. The number of genes annotated in each QTL region is in Table 4 and the gene names are in Table S2 (see Additional file 1: Table S2). Literature searches were carried out on the genes within each QTL region to identify those that were previously found to be associated with acute hyperthermia resistance. Meaningful candidate genes linked to acute hyperthermia resistance were found for QTL 13-1, 13-2, and 13-3 and will be discussed further.

### Comparison of TLE between genotypes at peak SNPs

We analysed the individual TLE of fish, corrected for the effects of dam and day, according to their genotypes at the peak SNP of each of the six identified QTL. Differences in TLE between the two homozygous genotypes ranged from 0.39 to 0.69 depending on the SNP (Table 5), representing between 39 and 69% of the phenotypic SD for TLE, as TLE was standardised to 1. As expected, the SNP for which the difference in TLE between the two homozygous genotypes was largest was located in TLE13-1, i.e. the QTL with the highest logBF. Boxplots of TLE corrected for the effects of day and dam depending on genotype at the peak SNP for each of the six QTL are in Additional file 2: Fig. S1.

**Table 5:** Differences in standardised acute hyperthermia resistance (TLE) and dominance effects for genotypes at the peak SNPs for the identified QTL

QTL name	Chr	Peak SNP identifier	Peak SNP position (Mb)	MAF	TLE difference between homozygous genotypes	Dominance effect (p-value)
TLE2-1	2	Affx-1237558532	78.97	0.34	0.39	0.00 (0.90)
TLE13-1	13	Affx-1237409861	36.68	0.42	0.69	− 0.05 (0.13)
TLE13-2	13	Affx-1237407727	45.63	0.46	0.37	0.09 **(0.01)**
TLE13-3	13	Affx-1237411204	47.37	0.24	0.49	− 0.08 **(0.04)**
TLE14-1	14	Affx-1237353641	42.93	0.31	0.41	− 0.04 (0.33)
TLE17-1	17	Affx-1248428573	20.73	0.21	0.45	0.02 (0.67)

Chr., chromosome; MAF, minor allele frequency; Mb stands for mega-base-pair; Dominance effect, mean difference between the heterozygous genotype TLE and the average of the two homozygous genotypes TLE, p-value in bold means the p-value is lower than the significance threshold of 0.05

Significant dominance effects were observed for genotypes at the peak SNP in TLE13-2 (p-value = 0.01) and TLE13-3 (p-value = 0.04) (Table 5).

### Refinement of the detected QTL using data from isogenic lines

Among the SNPs present on the HD genotyping array, we identified 52, 68, 13, 45, 5, and 0 SNPs in the TLE2-1, TLE13-1, -2, -3, TLE14-1, and TLE17-1 regions, respectively, for which alleles differed between the sensitive and the resistant isogenic lines. Of all these SNPs, the three that were located in TLE13-1 and TLE13-2 had a logBF ≥ 6 in the commercial population. Details about these SNPs, their effects in the commercial population, and the alleles of the resistant and sensitive lines are in Table 6. Consistently, the alleles at these three SNPs that were present in the resistant isogenic line were predicted by the GWAS of the commercial population to have a favourable effect on the acute hyperthermia resistance and vice-versa for the sensitive line.

**Table 6:** QTL shared between the commercial population and the isogenic lines

QTL name	Chr	SNP identifier	Pos (Mb)	Allele of the sensitive paternal line (A22)	Allele of the resistant paternal line (A32)	logBF in the commercial population	Favourable allele for TLE in the commercial population	Mean effect of the favourable allele for TLE in the commercial population (% of phenotypic standard deviation)
TLE13-1	13	Affx-1237410984	36.89	A	G	7.6	G	+ 15
TLE13-2	13	Affx-1237407727	45.64	G	T	6.1	T	+ 5
TLE13-2	13	Affx-1237407728	45.64	G	A	6.3	A	+ 6

Chr, chromosome; Pos, position on the Arlee genome assembly (GCA_013265735.3 USDA_OmykA_1.1)

Mb stands for mega-base-pair

## Discussion

In this study, we investigated the genetic architecture of resistance to acute hyperthermia in a French commercial population of rainbow trout at nine months of age. We estimated the genetic parameters of this trait and its correlations with production traits at a harvest weight of approximately 1 kg (body weight, fork length, carcass yield, and fillet fat percentage). We also identified QTL for resistance to acute hyperthermia and searched for functional candidate genes.

### Defining acute hyperthermia resistance

Under acute stress, exposure to the stressor is short and intense, while under chronic stress, exposure is weaker and repeated over time [45]. However, this definition is context-dependent [46]. For example, in rainbow trout, acute hyperthermia resistance has been defined as resistance to hyperthermia in a one-day challenge by Perry et al. [13], but in a 1-week challenge by Chen et al. [47]. Whether acute or chronic, response to a stressor involves different physiological mechanisms [45, 48, 49]. Although the correlation between acute and chronic resistance to temperature has rarely been studied, consistently, in rainbow trout and Atlantic salmon, no relationship has been found between resistance to acute hyperthermia, measured as temperature at loss of equilibrium within a day, and resistance to chronic hyperthermia, measured as growth or survival at high temperature during more than a month [50, 51]. To our knowledge, no research has been conducted on the relationship between acute resistance and hyperthermia stress at different levels of exposure and intensity, for example between a one-day hyperthermia challenge and a two-day hyperthermia challenge. Thus, a better characterisation of the acute hyperthermia resistance phenotype, particularly its genetic and phenotypic correlations with semi-chronic and chronic hyperthermia resistance, would be very valuable, as these stressful conditions can also occur in fish farms, but this is beyond the scope of the current study. Thus, in the following Discussion section, we considered that hyperthermia stress was acute only if fish lost equilibrium within a day, and to be semi-chronic or chronic otherwise.

### Phenotyping acute hyperthermia resistance

During acute hyperthermia resistance challenges, the parameters of water quality were carefully checked in the first three groups to ensure that they had only a limited impact on acute hyperthermia resistance of fish. It has been previously shown that O_2_ saturation does not interfere with resistance to acute hyperthermia in fish when O_2_ saturation is above 80%, which is the level maintained during acute hyperthermia phenotyping [52–54]. Given the pH (7.2 ± 0.4) and the short duration of the challenge, the ${\text{NH}}_{4}^{ + }$ concentration (less than 3 mg/L) remains under the toxicity threshold of rainbow trout [55]. In acute hyperthermia resistance phenotyping, CO_2_ concentration reached a maximum of 12 mg/L in the first 3 h. Chronic exposure to a two-fold higher CO_2_ concentration (24 ± 1 mg/L) during six months has been reported to have no significant impact on rainbow trout growth and survival [56]. Thus, increases in ${\text{NH}}_{4}^{ + }$ and CO_2_ concentrations were assumed to have minor effects on the resistance of fish to acute hyperthermia.

In the present paper, acute hyperthermia resistance was measured as the time to loss of equilibrium, while other studies have used cumulative degrees to quantify resistance to acute hyperthermia [13, 16, 57]. Cumulative degrees are calculated as a combination between time and temperature: the difference between temperature at each minute of the challenge and the initial temperature is cumulated from the beginning of the challenge to the time of loss of equilibrium [13]. The cumulative degrees phenotype is supposed to correct for differences in the rate of temperature increases between groups. However, in our study, there were still significant between-group differences in the mean (ANOVA, N = 1327, df = 6; F value = 359.69; P < 2.2e−16) and standard deviation (Levene's statistic, N = 1327, df = 6; F value = 57.459; P < 2.2e−16) for the cumulative degrees phenotype, similar to those for time to loss of equilibrium phenotypes. Cumulative degrees failed to homogenise the mean and standard deviation of the phenotyping groups. Moreover, the phenotypic correlation of cumulative degrees with time before loss of equilibrium was 0.98 and the estimate of the genetic correlation did not converge, as these two phenotypes were too highly correlated. This strong correlation might not be found in studies in which differences in temperature between phenotyping groups are larger than in our study. Because of the non-homogenization of phenotypes between groups and of this similarity between the two phenotypes, we decided to use the simplest phenotype, i.e. time to loss of equilibrium.

There were significant disparities between the phenotyping groups in terms of mean and SD of rTLE. The number of acclimation days before challenge and the temperature at six hours after the start of the challenge were strongly correlated with the means and SD of rTLE across groups, which may explain these disparities. However, because these two factors were confounded, it was impossible to determine which of them had a predominant effect on means and SD of acute hyperthermia of each group.

Acclimation, particularly the temperature that precedes acute hyperthermal stress, is a well-known factor that influences acute hyperthermia resistance of fish [10]. In our study, the first groups that were phenotyped were less acclimated to the temperature of the river (16–18 °C), which was higher than the temperature (11–12 °C) in the fish farm before acute hyperthermia resistance phenotyping and during transportation to the phenotyping platform (11–13 °C). As a result, these first groups had a lower mean for acute hyperthermia resistance. This was consistent with Chen et al. [58] who showed a strong positive correlation of 0.74 between acclimation temperature and acute hyperthermia resistance in rainbow trout.

The second factor that was identified to influence acute hyperthermia resistance of fish was the temperature after 6 h of challenge, which was highly correlated with means and SD of acute hyperthermia resistance of each group. Logically, phenotyping groups with a lower temperature after six hours of challenge resisted longer to acute hyperthermia stress, as in Becker et al. [59]. Slower temperature increases have also been shown to increase the dispersion (SD) of acute hyperthermia resistance phenotypes in ectotherms such as in the Cuyaba dwarf frog *Physalaemus* *nattereri,* although this varies between species [60]. In the present study, we consistently found that phenotyping groups that had a lower temperature after six hours of challenge had a higher mean and SD for acute hyperthermia resistance.

To account for the heterogeneity of variance among groups, group disparities were corrected by centring and scaling rTLE to a standard deviation of 1 for each challenge group (TLE) [61].

### Heritability estimates for acute hyperthermia resistance in juveniles and production traits

Pedigree- and genomic-based heritability estimates for acute hyperthermia resistance at 280 dpf were 0.24 ± 0.07 and 0.29 ± 0.05, respectively. These estimates are lower but consistent with an earlier study which reported a pedigree-based heritability of 0.41 ± 0.07 on a North-American population of commercial rainbow trout at an age between 148 and 300 dpf [13]. Our lower estimate of heritability is associated with the inclusion of a maternal effect that explains 6 to 7% of the TLE phenotypic variance. A maternal effect of similar magnitude was also detected for acute hypoxia resistance at 270 dpf in rainbow trout [62]. When the random maternal effect was ignored, the pedigree- and genomic-based estimates of heritability increased to 0.39 ± 0.06 and 0.35 ± 0.04, which are similar to the estimate of Perry et al. [13]. It is not clear whether the significance of the maternal effect was checked in Perry et al. [13].

The discovery of a significant maternal effect at an age as late as 280 dpf was surprising, since the magnitude of the maternal effect generally tends towards zero within the first year of life in fish for traits such as growth or survival [63, 64]. It appears that the magnitude of maternal effects on acute hyperthermia resistance also declines with age in salmonids: maternal effects were found to explain 77% of the acute hyperthermia resistance phenotypic variance in chinook salmon *Oncorhynchus* *tshawytscha* larvae weighing between 0.6 and 3.6 g [65], while it was not significant for acute hyperthermia resistance in Atlantic salmon at 297 dpf and a body weight of 35.0 ± 10.1 g [66].

In chinook salmon larvae, there was a strong correlation between the average egg diameter of females and the average acute hyperthermia resistance of their offspring, which suggests that the significant maternal effect could be due to the size of the eggs [65]. In the present study, since eggs were not sorted by size, it is possible that the maternal effect came from differences in mean egg size between females. Another hypothesis regarding the significant maternal effect could be the presence of maternal intergenerational plasticity effects and, more particularly their effects on mitochondria. Acclimation of dams to high temperatures has been shown to significantly affect the respiration capacity of the mitochondria of their offspring for up to at least 60 days in the stickleback *Gasterosteus* *aculeatus* [67]. The body weight of these offspring was influenced by the dam acclimation temperature, probably induced by different respiration capacities of the mitochondria [67]. It has also been shown in the stickleback that exposure of dams to warmer temperatures influences the mitochondrial DNA level in their oocytes, which significantly affects growth and mortality at least in the first week of life [68]. Thus, it is possible that maternal intergenerational plasticity could also impact traits such as acute hyperthermia resistance.

The heritability of BW1 was within the low range of previous estimates for BW at similar ages (0.20–0.28) [69, 70]. Heritability estimates for all other production traits were also consistent with those of a study on 17-month-old rainbow trout that originated from another French commercial population of rainbow trout [71].

### Genetic correlations of acute hyperthermia resistance in juveniles with production traits

Genetic correlations were estimated between acute hyperthermia resistance and production traits that are commonly selected for in fish breeding programs. The interest in estimating genetic correlations is twofold. First, from a biological point of view, genetic correlations can reveal the possible existence of shared biological pathways between traits [72]. Second, from a breeder's point of view, genetic correlations predict the effect of selecting one trait on responses for other traits of interest. Therefore, estimating genetic correlations is essential for describing the genetic architecture of a trait, as well as for optimisation of breeding programs.

The phenotypic correlation between TLE and BW1, both measured in batch B1 between 275 and 285 dpf, was slightly negative (− 0.07 ± 0.03), which is consistent with the literature, where the phenotypic relationship between BW and acute hyperthermia resistance was reported to be zero or negative in fish [73].

The genetic correlation between TLE and BW1 was clearly negative (− 0.49 ± 0.13). A previous study estimated a null genetic correlation (− 0.03 ± 0.18) between acute hyperthermia resistance and body weight at 210–259 dpf in a North American population of rainbow trout [13]. This difference between the two studies might be due to genetic or environmental differences between the studied populations, as was observed by Kause et al. [74] for body weight, different phenotyping ages, or separated or mixed family rearing. However, our result is consistent with that of Debes et al. [66] who, found a clearly negative genetic correlation of − 0.86 ± 0.49 between acute hyperthermia resistance and fork length in first-year Atlantic salmon; fork length is highly genetically correlated with body weight in Atlantic salmon [75] as well as in rainbow trout as shown in Table 3 and in Haffray et al. [30]. Trade-offs have been suggested between body weight and acute hyperthermia resistance in rainbow trout [11, 16] and in fish in general [73, 76], which suggests that some physiological mechanisms underlie this negative relationship that have a genetic basis. A controversial hypothesis to explain this link between body weight and acute hyperthermia resistance is the oxygen- and capacity-limitation of thermal tolerance. This theory states that the point of failure of acute hyperthermia resistance in fish is due to the inability of the organism to supply enough oxygen to the tissues as oxygen requirements increase exponentially with temperature [77]. Under this theory, larger fish are more sensitive to hyperthermia as their aerobic scope is reduced compared to smaller fish [77]. However, this theory failed to predict acute hyperthermia resistance in some fish species and thus cannot explain everything [78, 79]. We also examined whether there were some QTL that overlapped between TLE and BW1, which could explain part of the strong genetic correlation between these two traits. TLE13-1, the most evident QTL associated with acute hyperthermia resistance was found at the same position as a QTL related to BW that was previously detected in another population of rainbow trout at 410 to 481 dpf [80]. In our study, we detected one QTL for BW1 and 12 QTL for BW2 (see Additional file 1: Table S3 for the positions of the BW QTL). However, in our population, none of these QTL was at a similar position as that identified in Ali et al. [80] or overlapped with any of the QTL associated with TLE that we detected.

The estimate of the genetic correlation between TLE at nine months and body weight at 20 months was not significantly different from zero, which means that selecting for acute hyperthermia resistance at a juvenile age would have a limited impact on growth at the near harvest age and vice versa.

Two opposing hypotheses may explain why the estimate of the genetic correlation between TLE and BW2 was not significant while that between TLE and BW1 was significant. The first hypothesis is that acute hyperthermia resistance is not a stable trait between nine and 20 months in rainbow trout, i.e. that rankings of fish based on genetic resistance to acute hyperthermia can change with age. In that case, the genetic correlations estimated between production traits measured at 20 months of age and TLE measured at nine months of age might be poorly informative of the genetic correlations between production traits measured at 20 months and TLE measured at any other age than nine months. The genetic correlation between acute hyperthermia resistance at 20 months of age (not measured in our study) and BW2 might still be strongly negative, which would indicate a potential trade-off between growth and resistance to acute hyperthermia traits, as suggested by Roze et al. [11] in 1-year old rainbow trout. Thus, selecting for resistance to acute hyperthermia at nine months is not expected to improve resistance throughout the life cycle of the fish, which would reduce the value of selecting for this trait. However, this is not the most likely hypothesis, as several studies have shown good repeatability of acute hyperthermia resistance in brook trout *Salvelinus* *fontinalis* and rainbow trout over 1 year [16, 57, 81]. However, one study found no repeatability of acute hyperthermia resistance in sea bass *Dicentrarchus* *labrax* over 11 months [82], but this study was carried out in a semi-natural uncontrolled environment with probable strong genotype by environment interactions.

A second hypothesis would be that acute hyperthermia resistance remains stable with age and that the negative genetic correlation between acute hyperthermia resistance and body weight decreases with age in rainbow trout. The stability of acute hyperthermia resistance with age is consistent with the studies cited in the previous paragraph. The decreasing genetic correlation between acute hyperthermia resistance and BW with age is consistent with Lagarde et al. [16], who reported that body weight had a significant effect on acute hyperthermia resistance in rainbow trout at nine months, but no effect at 20 months. If this assumption is correct, selecting for acute hyperthermia resistance at any age would have no impact on growth at 20 months and vice versa.

Estimates of genetic correlations of TLE with the other production traits (FL, Fat% and HGCW) were also close to zero. Therefore, selecting for TLE at nine months likely will have limited impact on these traits and BW2 at 20 months. In addition, the selection for production traits that is currently performed in rainbow trout has probably not impaired its acute hyperthermia resistance.

### QTL for acute hyperthermia resistance in juveniles

We identified six QTL for acute hyperthermia resistance in a French commercial population of rainbow trout using a high-density chip with 665K SNPs, on chromosomes 2, 13, 14, and 17. Previous studies have identified QTL for acute hyperthermia resistance on chromosomes 1, 9, 19, and the sex chromosome Y in a North-American population of rainbow trout using a limited number of markers composed of allozymes, random amplification of polymorphic DNA (RAPD), and microsatellites [24–27, 83]. The absence of common QTL between our and previous studies is not surprising since the origins of the populations and the marker densities are very different [84] and the power to detect QTL of smaller effects is limited.

In total, the six QTL only explained 5% of the genetic variance of acute hyperthermia resistance (Table 4), of which the main QTL, TLE13-1, explained 4%. This suggests that resistance to acute hyperthermia is a highly polygenic trait in rainbow trout. Nevertheless, phenotypic differences depending on genotype at the peak SNP were substantial, with a mean TLE difference of up to 69% of the phenotypic standard deviation between the favourable and unfavourable homozygotes, which is considerable. In comparison, differences of 12 and 28% of the phenotypic standard deviation between homozygotes at peak SNPs for acute hyperthermia resistance were reported in the channel catfish and turbot *Scophthalmus* *maximus*, respectively [28, 85]. Thus, in spite of the low percentage of genetic variance explained at the population level, SNP peaks at the detected QTL seem to be good candidates for marker-assisted selection for acute hyperthermia resistance in the studied population.

In rainbow trout, previous studies have detected QTL for chronic hyperthermia resistance, i.e. the ability of fish to survive under chronic hyperthermia stress [47], and tolerance, i.e. the ability of fish to grow under chronic hyperthermia stress [86]. None of the QTL found in these two studies overlapped with the QTL identified here. This is expected since QTL tend to be population-specific, power to detect QTL of small effects is limited, and because resistance to acute hyperthermia and resistance or tolerance to chronic hyperthermia have been shown to be distinct traits in salmonids [50, 51].

### Functional candidate genes involved in acute hyperthermia resistance

In fish, GWAS for acute hyperthermia resistance were previously performed on the channel catfish [28] and the large yellow croaker [29], and identified 15 and 98 genes in the detected QTL, respectively. Among these genes, a gene from the *dnaj* gene family (also called *hsp40* gene family) was reported in the two studies: *dnajc25* in [28] and *dnajb4* in [29].

In line with these studies, we identified the *dnajc7* (*dnaJ* *heat* *shock* *protein* *family* *member* *C7*) gene at 150 kb from the SNP peak of the most significant QTL (TLE13-1). Dnaj proteins are co-chaperones of Hsp70 (heat shock protein 70) and play an important role in regulating the latter by recruiting Hsp70 partners and regulating the ATPase activity of the chaperone cycle [87]. In rainbow trout, genes of the *dnaj* family are overexpressed in several organs (heart, brain, liver, spleen) during acute hyperthermia stress [88, 89] and individuals that over-express *dnaj* genes are more resistant to acute heat stress than fish with a lower level of expression [90, 91]. These results suggest that *dnaj* is a key gene family for resistance to acute hyperthermia in rainbow trout and other fish species. Another member of the *dnaj* gene family has also been reported in a GWAS on chronic hyperthermia stress resistance in turbot, with an effect on time to loss of equilibrium greater than 1 week [85]. This result was surprising, as there is growing evidence that acute and chronic hyperthermia resistances are distinct traits, as previously mentioned in rainbow trout [50] and Atlantic salmon [51]. Nevertheless, it seems possible that some mechanisms of resistance to acute and chronic hyperthermia stress could be shared.

However, in the TLE13-1 region, we identified other promising functional candidate genes. Indeed, very close to the peak SNP (less than 100 kb), we identified the *hsp70b* (*heat* *shock* *protein* *70b*) gene and other *hsp70* homologues. The *hsp70* gene family has several functions, including molecular chaperoning and assisting the restoration or degradation of altered proteins [92]. The *hsp70* genes are commonly associated with protein folding in the generic response to stress exposure, especially acute hyperthermia exposure. In rainbow trout, larvae that show a strong ability to upregulate the *hsp70b* gene were found to be significantly more resistant to acute hyperthermia compared to those that did not have this ability [93]. *hsp70b* was also shown to be the most differentially expressed gene between a thermally selected strain and a thermal naïve strain of rainbow trout [91] and was found to be the most over-expressed gene of the *hsp* family during acute hyperthermia conditions in immortalised rainbow trout gonadal fibroblasts [94]. In other species, some genes from the *hsp70* family are also known to have a role in acute hyperthermia resistance. For example, in bay scallops *Argopecten* *irradians*, a polymorphism in the promoter of a *hsp70* family gene significantly affected acute hyperthermia resistance, with the genes being upregulated in the resistant individuals compared to the less resistant ones [95]. In the fruitfly *Drosophila* *buzzatii*, selection for heat resistance up to 64 generations has increased expression of the *hsp70* gene family [96].

The *dnajc7* and *hsp70* genes are only 15 kb apart and they have been shown to interact in similar pathways and notably during acute hyperthermia stress in mammals and fish [97–99]. These two properties suggest that these two genes may constitute a supergene associated with acute hyperthermia resistance, as previously identified for sex-specific migratory tendency in rainbow trout [100]. Supergenes are a group of segregating loci that provide integrated control of complex adaptive phenotypes [101]. In other words, we hypothesise that the *dnajc7* and *hsp70* genes have a role in acute hyperthermia resistance and that particularly favourable allelic combinations of these two genes may exist.

We identified four other potential candidate genes for QTL TLE13-1: *nkiras2*, *cdk12*, *phb*, and *fkbp10*. These genes may be related to resistance to acute hyperthermia, although with less supporting evidence than for *dnaj* and *hsp70*. The protein encoded by *nkiras2* (NF-κB-inhibitor-interacting Ras 2) regulates NF-κB factors, which are involved in the homeostasis maintenance in mammalian cells [102] and in increasing the transduction of genes that have a role in the inflammatory response in rainbow trout [103]. The *nkiras2* gene is upregulated in gills of the chinook salmon exposed to acute hyperthermia stress (12 °C to 25 °C during three hours) [104]. The *cdk12* (*cyclin* *dependent* *kinase* *12*) gene encodes a Pol II CTD kinase. In the fruitfly *Drosophila* *melanogaster*, cdk12 is known to be involved in the control of the transcription of a set of genes that have a role in the response to various stress factors: heat shock [105], DNA damage [106], and oxidative stress [107]. A lack of function of cdk12 was shown to increase the sensitivity of flies to oxidative stress [107]. The *phb* (*prohibitin*) gene encodes a highly conserved protein with roles in many diverse functions such as chaperoning activities in the mitochondria [108], the activation of transcription signalling pathways [109] or the regulation of cell survival and apoptosis [110]. This gene is upregulated under acute heat stress in the salt marsh mussel *Geukensia* *demissa* [111] and in a cell line derived from a human tongue squamous cell carcinoma [112]. These two studies, however, disagree on the presumed role of the prohibitin protein: Fields et al. [111] hypothesised that the prohibitin level increased in cells to delay or prevent heat-induced apoptosis, while Jiang et al. [112] argued that the increased abundance of prohibitin in cells may promote their apoptosis. Since prohibitin has many functions, some of which are poorly understood, it is difficult to hypothesize what effect this protein might have on resistance to acute hyperthermia in rainbow trout. The *fkbp10* (*FKBP* *prolyl* *isomerase* *10*) gene is part of the FK506-binding protein gene family, involved in multiple functions including protein folding and repairing [113]. It is downregulated in salmonids that are subjected to chronic heat stress [114] but, to date, no study has reported a differential expression of *fkbp10* during acute hyperthermia stress in fish.

In the isogenic lines, we found that the line, which was more resistant to acute hyperthermia, carried an allele at position 36.892 Mb on chromosome 13 that was predicted to improve the resistance to acute hyperthermia in the commercial population, while the sensitive isogenic line carried the deleterious allele. The QTL TLE13-1 may be shared between the commercial population and the isogenic lines, which represent two populations of rainbow trout that are separated by a moderately short genetic distance. Indeed, the fixation index (Fst) between the commercial population and the INRAE synthetic line from which the isogenic lines were derived was estimated to be 0.09 [115]. The information obtained from the whole-genome sequencing of the isogenic lines refines the likely position of the TLE13-1 QTL candidate genes to be close to 36.892 Mb on chromosome 13. This confirms that genes near this position (the *hsp70* gene family 36.744–36.827 Mb, *dnajc7* 36.842–36.851 Mb and *nkiras2* 36.851–36.853 Mb) are highly plausible functional candidate genes.

For the TLE13-2 QTL, we identified two interesting genes that were previously shown to be differentially expressed during acute hyperthermia exposure in fish: *ddx5* (*probable* *ATP-dependent* *RNA* *helicase* *ddx5*) and *cygb1*(*cytoglobin-1*). After acute hyperthermia stress, the *ddx5* gene was shown to be significantly downregulated in the salmonid taimen *Hucho* *taimen* [116] and the *cygb1* gene was shown to be significantly upregulated in channel catfish [117]. The resistant isogenic line was found to carry two alleles that were predicted to increase TLE in the commercial population. These two alleles are located at positions 45.638 and 45.640 Mb, which are closer to the location of the *ddx5* gene (45.622–45.626 Mb) than to that of *cygb1* (45.711–45.717 Mb).

For the TLE13-3 QTL, the gene *enpp7* (*ectonucleotide* *pyrophosphatase/phosphodiesterase* *family* *member* *7*) was close to the peak SNP. The protein encoded by the *enpp7* gene is an alkaline sphingomyelinase that hydrolyses membrane sphingomyelin to ceramide and phosphocholine [118]. We found no direct or indirect link between the function of enpp7 and acute hyperthermia resistance but a GWAS of acute hyperthermia tolerance in pacific abalone *Haliotis* *discus* also identified this gene to be in a QTL region [119], which supports a possible role of this gene in acute hyperthermia resistance.

Last but not least, seven nuclear genes among the candidate genes were found to encode proteins with strong support for mitochondrial localisation in humans according to the Human MitoCarta3.0 database (https://www.broadinstitute.org/mitocarta). These genes, some of which have already been discussed, were *pdhx* (*pyruvate* *dehydrogenase* *protein* *X*) (TLE-2-1), *phb*, *fkbp10*, *acly* (*ATP* *citrate* *lyase*), *hsp70* (TLE-13-1), *elac2,* and *sco1* (TLE-13–3). One reason for the lack of acute hyperthermia resistance in fish may be related to the disrupted ability of mitochondria to produce ATP at high temperatures, although evidence for this is still limited [120]. Interestingly, the *pdhx* and *acly* genes are involved in ATP anabolic or catabolic processes. The *pdhx* gene is part of the pyruvate dehydrogenase complex, which catalyses the oxidative decarboxylation of pyruvate into acetyl-CoA, a reaction that notably ensures the link between glycolysis and the Krebs cycle [121, 122]. The *acly* encodes an enzyme that is involved in fatty acid biosynthesis, which generates acetyl-CoA from citrate by consuming ATP [123, 124]. This also strengthens the previously mentioned hypothesis that the significant maternal effect found for TLE is related to mitochondria.

## Conclusions

This work provides new insights into the genetic architecture of acute hyperthermia resistance in rainbow trout juveniles using a novel high-density genotyping array with 665K SNPs. Heritability of acute hyperthermia resistance at a juvenile stage was moderate and its genetic correlations with the main production traits measured in sibs at harvest age were estimated to be close to zero. Incorporating a trait for resistance to acute hyperthermia in selection programs would make it possible to obtain robust animals without reducing production performance. However, this should be confirmed by ensuring that the genetic correlation between TLE at the juvenile stage and at harvest age is high. Our study demonstrates that acute hyperthermia resistance is polygenic in rainbow trout, which is consistent with observations in other fish species. Indeed, the six identified QTL only explained around 5% of the genetic variance. The most significant QTL, located on chromosome 13, explained 4% of the genetic variance and genes directly associated with acute hyperthermia resistance (*hsp70* genes family and *dnajc7*) were located close to the peak SNP for this QTL. The identified QTL also contained genes associated with protein chaperoning, oxidative stress response, homeostasis maintenance, and cell survival, and are thus good candidates for further functional validation. As a preliminary validation of the detected QTL, we investigated the genotypes of two isogenic lines with contrasting resistance to acute hyperthermia. The resistant isogenic line was shown to carry favourable alleles in the region of two (including the main QTL) of the six QTL, which suggests that these QTL may be shared between distinct populations. In spite of the polygenic architecture of acute hyperthermia resistance, the difference in average TLE between homozygotes at SNP peaks for the QTL was large, which indicates great potential for marker-assisted selection. All these results demonstrate the relevance of selective breeding to improve fish acute hyperthermia resistance.

## Supplementary Information

**Additional file 1: Table S1**. Heritability and genetic correlations estimated under pedigree BLUP models. Heritability estimates are in bold on the diagonal, genetic correlations are in the upper triangle for resistance to acute hyperthermia measured as time to loss of equilibrium centred and reduced by group of challenge; body weight of batch 1; body weight of batch 2; fork length; fillet fat percentage; headed gutted carcass yield. All values are given with their standard error. **Table S2**.  Candidate genes from the NCBI Oncorhynchus mykiss Annotation Release 100that were located within the QTL regions. **Table S3.** QTL of body weight in B1and B2. Description: Chr: chromosome; #, number; % variance explained by QTL, % of genetic variance explained by all the SNPs included in the QTL region.

**Additional file 2**: **Figure S1.** Boxplots of the centred and reduced acute hyperthermia resistance corrected for day and dam effects depending on the genotypes of the 1328 fish at the peak SNPs of the six detected QTL. The Y-axis represents the TLE of fish and the three colored boxes represent the three genotypes for a given SNP. The dots represent the fish individual phenotype.

## Data Availability

The data used for this research are not publicly available.

## References

[1] Boyd CE, McNevin AA, Davis RP (2022). The contribution of fisheries and aquaculture to the global protein supply. Food Secur.

[2] FAO. The State of World Fisheries and Aquaculture 2022. Towards Blue Transformation. Rome: FAO; 2022. https://www.fao.org/3/ca9229en/online/ca9229en.html#chapter-1_1.

[3] Maulu S, Hasimuna OJ, Haambiya LH, Monde C, Musuka CG, Makorwa TH (2021). Climate change effects on aquaculture production: sustainability implications, mitigation, and adaptations. Front Sustain Food Syst.

[4] Alfonso S, Gesto M, Sadoul B (2021). Temperature increase and its effects on fish stress physiology in the context of global warming. J Fish Biol.

[5] Vasseur DA, DeLong JP, Gilbert B, Greig HS, Harley CDG, McCann KS (2014). Increased temperature variation poses a greater risk to species than climate warming. Proc R Soc B Biol Sci.

[6] Sandblom E, Clark TD, Gräns A, Ekström A, Brijs J, Sundström LF (2016). Physiological constraints to climate warming in fish follow principles of plastic floors and concrete ceilings. Nat Commun.

[7] Wade NM, Clark TD, Maynard BT, Atherton S, Wilkinson RJ, Smullen RP (2019). Effects of an unprecedented summer heatwave on the growth performance, flesh colour and plasma biochemistry of marine cage-farmed Atlantic salmon (*Salmo*
*salar*). J Therm Biol.

[8] Reid GK, Gurney-Smith HJ, Flaherty M, Garber AF, Forster I, Brewer-Dalton K (2019). Climate change and aquaculture: considering adaptation potential. Aquac Environ Interact.

[9] Pettinau L, Seppänen E, Sikanen A, Anttila K (2022). Aerobic exercise training with optimal intensity increases cardiac thermal tolerance in juvenile Rainbow trout. Front Mar Sci.

[10] Beitinger TL, Bennett WA, Mccauley RW (2000). Temperature tolerances of North American freshwater fishes exposed to dynamic changes in temperature. Environ Biol Fishes.

[11] Roze T, Christen F, Amerand A, Claireaux G (2013). Trade-off between thermal sensitivity, hypoxia tolerance and growth in fish. J Therm Biol.

[12] Falconer DS (1962). Introduction to quantitative genetics.

[13] Perry GML, Martyniuk CM, Ferguson MM, Danzmann RG (2005). Genetic parameters for upper thermal tolerance and growth-related traits in rainbow trout (*Oncorhynchus*
*mykiss*). Aquaculture.

[14] Morgan R, Finnøen MH, Jensen H, Pélabon C, Jutfelt F (2020). Low potential for evolutionary rescue from climate change in a tropical fish. Proc Natl Acad Sci USA.

[15] Chavanne H, Janssen K, Hofherr J, Contini F, Haffray P, Aquatrace Consortium (2016). A comprehensive survey on selective breeding programs and seed market in the European aquaculture fish industry. Aquac Int..

[16] Lagarde H, Phocas F, Pouil S, Goardon L, Bideau M, Guyvarc’h F (2023). Are resistances to acute hyperthermia or hypoxia stress similar and consistent between early and late ages in rainbow trout using isogenic lines?. Aquaculture.

[17] Wang H, Misztal I, Aguilar I, Legarra A, Muir WM (2012). Genome-wide association mapping including phenotypes from relatives without genotypes. Genet Res (Camb).

[18] Ren D, An L, Li B, Qiao L, Liu W (2021). Efficient weighting methods for genomic best linear-unbiased prediction (BLUP) adapted to the genetic architectures of quantitative traits. Heredity (Edinb).

[19] Zhou Q, Chen YD, Lu S, Liu Y, Xu WT, Li YZ (2021). Development of a 50K SNP array for Japanese flounder and its application in genomic selection for disease resistance. Eengineering.

[20] Fraslin C, Koskinen H, Nousianen A, Houston RD, Kause A (2022). Genome-wide association and genomic prediction of resistance to * Flavobacterium *
* columnare * in a farmed rainbow trout population. Aquaculture.

[21] Houston RD, Haley CS, Hamilton A, Guy DR, Tinch AE, Taggart JB (2008). Major quantitative trait loci affect resistance to infectious pancreatic necrosis in Atlantic salmon (*Salmo*
*salar*). Genetics.

[22] Moen T, Baranski M, Sonesson AK, Kjøglum S (2009). Confirmation and fine-mapping of a major QTL for resistance to infectious pancreatic necrosis in Atlantic salmon (*Salmo*
*salar*): population-level associations between markers and trait. BMC Genomics.

[23] Yue GH (2014). Recent advances of genome mapping and marker-assisted selection in aquaculture. Fish Fish.

[24] Jackson TR, Ferguson MM, Danzmann RG, Fishback AG, Ihssen PE, O’Connell M (1998). Identification of two QTL influencing upper temperature tolerance in three rainbow trout (*Oncorhynchus*
*mykiss*) half-sib families. Heredity (Edinb).

[25] Danzmann RG, Jackson TR, Ferguson MM (1999). Epistasis in allelic expression at upper temperature tolerance QTL in rainbow trout. Aquaculture.

[26] Perry GML, Danzmann RG, Ferguson MM, Gibson JP (2001). Quantitative trait loci for upper thermal tolerance in outbred strains of rainbow trout (*Oncorhynchus*
*mykiss*). Heredity (Edinb).

[27] Perry GML, Ferguson MM, Sakamoto T, Danzmann RG (2005). Sex-linked quantitative trait loci for thermotolerance and length in the rainbow trout. J Hered.

[28] Jin Y, Zhou T, Geng X, Liu S, Chen A, Yao J (2017). A genome-wide association study of heat stress-associated SNPs in catfish. Anim Genet.

[29] Wu Y, Zhou Z, Pan Y, Zhao J, Bai H, Chen B (2021). GWAS identified candidate variants and genes associated with acute heat tolerance of large yellow croaker. Aquaculture.

[30] Haffray P, Bugeon J, Rivard Q, Quittet B, Puyo S, Allamelou JM (2013). Genetic parameters of in-vivo prediction of carcass, head and fillet yields by internal ultrasound and 2D external imagery in large rainbow trout (*Oncorhynchus*
*mykiss*). Aquaculture.

[31] Haffray P, Bugeon J, Pincent C, Chapuis H, Mazeiraud E, Rossignol MN (2012). Negative genetic correlations between production traits and head or bony tissues in large all-female rainbow trout (*Oncorhynchus*
*mykiss*). Aquaculture.

[32] Douirin C, Haffray P, Vallet JL, Fauconneau B (1998). Determination of the lipid content of rainbow trout (*Oncorhynchus*
*mykiss*) filets with the torry fish fat meter R. Sci Aliment.

[33] Palti Y, Gao G, Liu S, Kent MP, Lien S, Miller MR (2015). The development and characterization of a 57K single nucleotide polymorphism array for rainbow trout. Mol Ecol Resour.

[34] Bernard M, Dehaullon A, Gao G, Paul K, Lagarde H, Prchal M (2022). Development of a high-density 665 K SNP array for rainbow trout genome-wide genotyping. Front Genet.

[35] Gao G, Magadan S, Waldbieser GC, Youngblood RC, Wheeler PA, Scheffler BE (2021). A long reads-based de-novo assembly of the genome of the Arlee homozygous line reveals chromosomal rearrangements in rainbow trout. G3 (Bethesda)..

[36] Griot R, Allal F, Brard-Fudulea S, Morvezen R, Haffray P, Phocas F (2020). APIS: an auto-adaptive parentage inference software that tolerates missing parents. Mol Ecol Resour.

[37] Sargolzaei M, Chesnais JP, Schenkel FS (2014). A new approach for efficient genotype imputation using information from relatives. BMC Genomics.

[38] Misztal I, Tsuruta S, Strabel T, Auvray B, Druet T, Lee DH. Blupf90 and related programs (Bgf90). In: Proceedings of the7th World Congress on Genetics Applied to Livestock Production: 19–23 August 19–23 2002; Montpellier. 2002.

[39] VanRaden PM (2008). Efficient methods to compute genomic predictions. J Dairy Sci.

[40] Habier D, Fernando RL, Kizilkaya K, Garrick DJ (2011). Extension of the bayesian alphabet for genomic selection. BMC Bioinformatics.

[41] Boerner V, Tier B (2016). BESSiE: a software for linear model BLUP and Bayesian MCMC analysis of large-scale genomic data. Genet Sel Evol BioMed Central.

[42] Kass RE, Raftery AE (1995). Bayes factors. J Am Stat Assoc.

[43] Michenet A, Barbat M, Saintilan R, Venot E, Phocas F (2016). Detection of quantitative trait loci for maternal traits using high-density genotypes of Blonde d’Aquitaine beef cattle. BMC Genet.

[44] Quillet E, Dorson M, Le Guillou S, Benmansour A, Boudinot P (2007). Wide range of susceptibility to rhabdoviruses in homozygous clones of rainbow trout. Fish Shellfish Immunol.

[45] Wendelaar Bonga SE (1997). The stress response in fish. Physiol Rev.

[46] Schreck CB, Tort L (2016). The concept of stress in fish. Fish Physiol.

[47] Chen Z, Narum SR (2021). Whole genome resequencing reveals genomic regions associated with thermal adaptation in redband trout. Mol Ecol.

[48] Barton BA (2002). Stress in fishes: a diversity of responses with particular reference to changes in circulating corticosteroids. Integr Comp Biol.

[49] Tort L (2011). Stress and immune modulation in fish. Dev Comp Immunol.

[50] Dupont-Nivet M, Crusot M, Rigaudeau D, Quillet E. Genetic analysis of resistance to acute or chronic temperature stress using isogenic lines of Rainbow trout (*Oncorhynchus**mykiss*). In: Proceedings of the 10th World Congress on Genetics Applied to Livestock Production: 17–22 August 2014; Vancouver; 2014.

[51] Bartlett CB, Garber AF, Gonen S, Benfey TJ (2022). Acute critical thermal maximum does not predict chronic incremental thermal maximum in Atlantic salmon ( * Salmo *
* salar * ). Comp Biochem Physiol Part A Mol Integr Physiol.

[52] Wang T, Lefevre S, Iversen NK, Findorf I, Buchanan R, Mckenzie DJ (2014). Anaemia only causes a small reduction in the upper critical temperature of sea bass: is oxygen delivery the limiting factor for tolerance of acute warming in fishes?. J Exp Biol.

[53] Brijs J, Jutfelt F, Clark TD, Gräns A, Ekström A, Sandblom E (2015). Experimental manipulations of tissue oxygen supply do not affect warming tolerance of European perch. J Exp Biol.

[54] Ern R, Norin T, Gamperl AK, Esbaugh AJ (2016). Oxygen dependence of upper thermal limits in fishes. J Exp Biol.

[55] Thurston RV, Russo RC, Vinogradov GA (1981). Ammonia toxicity to fishes. Effect of pH on the toxicity of the un-ionized ammonia species. Environ Sci Technol.

[56] Good C, Davidson J, Welsh C, Snekvik K, Summerfelt S (2010). The effects of carbon dioxide on performance and histopathology of rainbow trout *Oncorhynchus*
*mykiss* in water recirculation aquaculture systems. Aquac Eng.

[57] O’Donnell MJ, Regish AM, McCormick SD, Letcher BH (2020). How repeatable is CTmax within individual brook trout over short- and long-time intervals?. J Therm Biol.

[58] Chen Z, Snow M, Lawrence CS, Church AR, Narum SR, Devlin RH (2015). Selection for upper thermal tolerance in rainbow trout (*Oncorhynchus*
*mykiss* Walbaum). J Exp Biol.

[59] Becker CD, Genoway RG (1979). Evaluation of the critical thermal maximum for determining thermal tolerance of freshwater fish. Environ Biol Fishes.

[60] Agudelo-Cantero GA, Navas CA (2019). Interactive effects of experimental heating rates, ontogeny and body mass on the upper thermal limits of anuran larvae. J Therm Biol.

[61] Hill WG (1984). On selection among groups with heterogeneous variance. Anim Prod.

[62] Prchal M, D’Ambrosio J, Lagarde H, Lallias D, Patrice P, François Y (2023). Genome-wide association study and genomic prediction of hypoxia stress tolerance in rainbow trout. Aquaculture.

[63] Heath DD, Fox CW, Heath JW (1999). Maternal effects on offspring size: variation through early development of chinook salmon. Evolution (N Y).

[64] Lindholm AK, Hunt J, Brooks R (2006). Where do all the maternal effects go? Variation in offspring body size through ontogeny in the live-bearing fish *Poecilia*
*parae*. Biol Lett.

[65] Muñoz NJ, Anttila K, Chen Z, Heath JW, Farrell AP, Neff BD (2014). Indirect genetic effects underlie oxygenlimited thermal tolerance within a coastal population of chinook salmon. Proc R Soc B Biol Sci.

[66] Debes PV, Solberg MF, Matre IH, Dyrhovden L, Glover KA (2021). Genetic variation for upper thermal tolerance diminishes within and between populations with increasing acclimation temperature in Atlantic salmon. Heredity (Edinb).

[67] Shama LNS, Strobel A, Mark FC, Wegner KM (2014). Transgenerational plasticity in marine sticklebacks: maternal effects mediate impacts of a warming ocean. Funct Ecol.

[68] Kim SY, Chiara V, Álvarez-Quintero N, Velando A (2022). Mitochondrial DNA content in eggs as a maternal effect. Proc Biol Sci.

[69] Gall GAE, Huang N (1988). Heritability and selection schemes for rainbow trout: body weight. Aquaculture.

[70] Leeds TD, Vallejo RL, Weber GM, Gonzalez-Pena D, Silverstein JT (2016). Response to five generations of selection for growth performance traits in rainbow trout (*Oncorhynchus*
*mykiss*). Aquaculture.

[71] Blay C, Haffray P, Bugeon J, D’Ambrosio J, Dechamp N, Collewet G (2021). Genetic parameters and genome-wide association studies of quality traits characterised using imaging technologies in Rainbow trout, * Oncorhynchus mykiss*. Front Genet.

[72] van Rheenen W, Peyrot WJ, Schork AJ, Lee SH, Wray NR (2019). Genetic correlations of polygenic disease traits: from theory to practice. Nat Rev Genet.

[73] McKenzie DJ, Zhang Y, Eliason EJ, Schulte PM, Claireaux G, Blasco FR (2021). Intraspecific variation in tolerance of warming in fishes. J Fish Biol.

[74] Kause A, Mäntysaari E, Ritola O, Paananen T, Eskelinen U (2002). Coupling body weight and its composition: a quantitative genetic analysis in rainbow trout. Aquaculture.

[75] Refstie T, Steine TA (1978). Selection experiments with salmon III. Genetic and environmental sources of variation in length and weight of Atlantic salmon in the freshwater phase. Aquaculture.

[76] Recsetar MS, Zeigler MP, Ward DL, Bonar SA, Caldwell CA (2012). Relationship between fish size and upper thermal tolerance. Trans Am Fish Soc.

[77] Pörtner HO, Knust R (2007). Climate change affects marine fishes through the oxygen limitation of thermal tolerance. Science.

[78] Norin T, Malte H, Clark TD (2014). Aerobic scope does not predict the performance of a tropical eurythermal fish at elevated temperatures. J Exp Biol.

[79] Jutfelt F, Norin T, Ern R, Overgaard J, Wang T, McKenzie DJ (2018). Oxygen- and capacity-limited thermal tolerance: blurring ecology and physiology. J Exp Biol.

[80] Ali A, Al-Tobasei R, Lourenco D, Leeds T, Kenney B, Salem M (2020). Genome-wide identification of loci associated with growth in rainbow trout. BMC Genomics.

[81] Strowbridge N, Northrup SL, Earhart ML, Blanchard TS, Schulte PM (2021). Acute measures of upper thermal and hypoxia tolerance are not reliable predictors of mortality following environmental challenges in rainbow trout (*Oncorhynchus*
*mykiss*). Conserv Physiol..

[82] Mauduit F, Domenici P, Farrell AP, Lacroix C, Le Floch S, Lemaire P (2016). Assessing chronic fish health: an application to a case of an acute exposure to chemically treated crude oil. Aquat Toxicol.

[83] Perry GML, Ferguson MM, Danzmann RG (2003). Effects of genetic sex and genomic background on epistasis in rainbow trout (*Oncorhynchus*
*mykiss*). Genetica.

[84] Tsai HY, Hamilton A, Guy DR, Tinch AE, Bishop SC, Houston RD (2015). The genetic architecture of growth and fillet traits in farmed Atlantic salmon (*Salmo*
*salar*). BMC Genet.

[85] Ma A, Huang Z, X-an W, Xu Y, Guo X (2021). Identification of quantitative trait loci associated with upper temperature tolerance in turbot, *Scophthalmus*
*maximus*. Sci Rep.

[86] Yoshida GM, Yáñez JM (2021). Increased accuracy of genomic predictions for growth under chronic thermal stress in rainbow trout by prioritizing variants from GWAS using imputed sequence data. Evol Appl..

[87] Kelley WL (1998). The J-domain family and the recruitment of chaperone power. Trends Biochem Sci.

[88] Li Y, Huang J, Liu Z, Zhou Y, Xia B, Wang Y (2017). Transcriptome analysis provides insights into hepatic responses to moderate heat stress in the rainbow trout (*Oncorhynchus*
*mykiss*). Gene.

[89] Li Z, Liu Z, Wang YN, Kang YJ, Wang JF, Shi HN (2016). Effects of heat stress on serum cortisol, alkaline phosphatase activity and heat shock protein 40 and 90β mRNA expression in rainbow trout *Oncorhynchus*
*mykiss*. Biologia.

[90] Ojima N, Mekuchi M, Ineno T, Tamaki K, Kera A, Kinoshita S (2012). Differential expression of heat-shock proteins in F2 offspring from F1 hybrids produced between thermally selected and normal rainbow trout strains. Fish Sci.

[91] Tan E, Wongwarangkana C, Kinoshita S, Suzuki Y, Oshima K, Hattori M (2012). Global gene expression analysis of gill tissues from normal and thermally selected strains of rainbow trout. Fish Sci.

[92] Mayer MP, Bukau B (2005). Hsp70 chaperones: cellular functions and molecular mechanism. Cell Mol Life Sci.

[93] Blair SD, Glover CN (2019). Acute exposure of larval rainbow trout (*Oncorhynchus*
*mykiss*) to elevated temperature limits hsp70b expression and influences future thermotolerance. Hydrobiologia.

[94] Ojima N, Yamashita M, Watabe S (2005). Quantitative mRNA expression profiling of heat-shock protein families in rainbow trout cells. Biochem Biophys Res Commun.

[95] Yang C, Wang L, Wang J, Jiang Q, Qiu L, Zhang H (2014). The polymorphism in the promoter of HSP70 gene is associated with heat tolerance of two congener endemic bay scallops ( * Argopecten *
* irradians *
* irradians * and * A. *
* i *
* . *
* concentricus * ). PLoS ONE.

[96] Sørensen JG, Michalak P, Justesen J, Loeschcke V (1999). Expression of the heat-shock protein HSP70 in *Drosophila*
*buzzatii* lines selected for thermal resistance. Hereditas.

[97] Ohtsuka K, Hata M (2000). Molecular chaperone function of mammalian Hsp70 and Hsp40—a review. Int J Hyperth.

[98] Qiu XB, Shao YM, Miao S, Wang L (2006). The diversity of the DnaJ/Hsp40 family, the crucial partners for Hsp70 chaperones. Cell Mol Life Sci.

[99] Li J, Zhang Y, Liu Y, Zhang Y, Xiao S, Yu Z (2016). Co-expression of heat shock protein (HSP) 40 and HSP70 in *Pinctada*
*martensii* response to thermal, low salinity and bacterial challenges. Fish Shellfish Immunol.

[100] Pearse DE, Barson NJ, Nome T, Gao G, Campbell MA, Abadía-Cardoso A (2019). Sex-dependent dominance maintains migration supergene in rainbow trout. Nat Ecol Evol.

[101] Joron M, Frezal L, Jones RT, Chamberlain NL, Lee SF, Haag CR (2011). Chromosomal rearrangements maintain a polymorphic supergene controlling butterfly mimicry. Nature.

[102] Oeckinghaus A, Postler TS, Rao P, Schmitt H, Schmitt V, Grinberg-Bleyer Y (2014). κB-Ras proteins regulate both NF-κB-dependent inflammation and Ral-dependent proliferation. Cell Rep.

[103] Sarais F, Rebl H, Verleih M, Ostermann S, Krasnov A, Köllner B (2020). Characterisation of the teleostean κB-Ras family: the two members NKIRAS1 and NKIRAS2 from rainbow trout influence the activity of NF-κB in opposite ways. Fish Shellfish Immunol.

[104] Tomalty KMH, Meek MH, Stephens MR, Rincón G, Fangue NA, May BP (2015). Transcriptional response to acute thermal exposure in juvenile Chinook salmon determined by RNAseq. G3 (Bethesda)..

[105] Bartkowiak B, Liu P, Phatnani HP, Fuda NJ, Cooper JJ, Price DH (2010). CDK12 is a transcription elongation-associated CTD kinase, the metazoan ortholog of yeast Ctk1. Genes Dev.

[106] Blazek D, Kohoutek J, Bartholomeeusen K, Johansen E, Hulinkova P, Luo Z (2011). The cyclin K/Cdk12 complex maintains genomic stability via regulation of expression of DNA damage response genes. Genes Dev.

[107] Li X, Chatterjee N, Spirohn K, Boutros M, Bohmann D (2016). Cdk12 is a gene-selective RNA polymerase II kinase that regulates a subset of the transcriptome, including Nrf2 target genes. Sci Rep.

[108] Nijtmans LGJ, Artal Sanz M, Grivell LA, Coates PJ (2002). The mitochondrial PHB complex: roles in mitochondrial respiratory complex assembly, ageing and degenerative disease. Cell Mol Life Sci.

[109] Mishra S, Ande SR, Nyomba BLG (2010). The role of prohibitin in cell signaling. FEBS J.

[110] Peng YT, Chen P, Ouyang RY, Song L (2015). Multifaceted role of prohibitin in cell survival and apoptosis. Apoptosis.

[111] Fields PA, Burmester EM, Cox KM, Karch KR (2016). Rapid proteomic responses to a near-lethal heat stress in the salt marsh mussel *Geukensia*
*demissa*. J Exp Biol.

[112] Jiang W, Bian L, Wang N, He Y (2013). Proteomic analysis of protein expression profiles during hyperthermia-induced apoptosis in Tca8113 cells. Oncol Lett.

[113] Tong M, Jiang Y (2015). FK506-binding proteins and their diverse functions. Curr Mol Pharmacol.

[114] Akbarzadeh A, Günther OP, Houde AL, Li S, Ming TJ, Jeffries KM (2018). Developing specific molecular biomarkers for thermal stress in salmonids. BMC Genomics.

[115] D’Ambrosio J, Phocas F, Haffray P, Bestin A, Brard-Fudulea S, Poncet C (2019). Genome-wide estimates of genetic diversity, inbreeding and effective size of experimental and commercial rainbow trout lines undergoing selective breeding. Genet Sel Evol.

[116] Liu Y, Muniz MMM, Lam S, Song D, Zhang Y, Yin J (2021). Gene expression profile of the taimen * Hucho *
* taimen * in response to acute temperature changes. Comp Biochem Physiol Part D Genomics Proteomics.

[117] Feng JB, Liu SK, Wang RJ, Zhang JR, Wang XL, Kaltenboeck L (2015). Molecular characterization, phylogenetic analysis and expression profiling of myoglobin and cytoglobin genes in response to heat stress in channel catfish *Ictalurus*
*punctatus*. J Fish Biol.

[118] Borza R, Salgado-Polo F, Moolenaar WH, Perrakis A (2022). Structure and function of the ecto-nucleotide pyrophosphatase/phosphodiesterase (ENPP) family: tidying up diversity. J Biol Chem.

[119] Yu F, Peng W, Tang B, Zhang Y, Wang Y, Gan Y (2021). A genome-wide association study of heat tolerance in Pacific abalone based on genome resequencing. Aquaculture.

[120] Chung DJ, Schulte PM (2020). Mitochondria and the thermal limits of ectotherms. J Exp Biol.

[121] Patel MS, Roche TE (1990). Molecular biology and biochemistry of pyruvate dehydrogenase complexes. FASEB J.

[122] Patel MS, Nemeria NS, Furey W, Jordan F (2014). The pyruvate dehydrogenase complexes: structure-based function and regulation. J Biol Chem.

[123] Chypre M, Zaidi N, Smans K (2012). ATP-citrate lyase: a mini-review. Biochem Biophys Res Commun.

[124] Feng X, Zhang L, Xu S, A-zong S (2020). ATP-citrate lyase (ACLY) in lipid metabolism and atherosclerosis: an updated review. Prog Lipid Res.

