# Supplementary material for: Genetic architecture of acute hyperthermia resistance in juvenile rainbow trout (Oncorhynchus mykiss) and genetic correlations with production traits
Source: Genet Sel Evol. 2023 Jun 12;55:39. doi: 10.1186/s12711-023-00811-4 (PMC10259007; doi:10.1186/s12711-023-00811-4)
Supplement: Supplementary file 2 — Additional file 2: Figure S1. Boxplots of the centred and reduced acute hyperthermia resistance corrected for day and dam effects depending on the genotypes of the 1328 fish at the peak SNPs of the six detected QTL. The Y-axis represents the TLE of fish and the three colored boxes represent the three genotypes for a given SNP. The dots represent the fish individual phenotype. [file 12711_2023_811_MOESM2_ESM.docx]

**Additional file 2 Figure S1**

Boxplots of the centred and reduced acute hyperthermia resistance corrected from day and dam effects depending on the genotypes of the 1,328 fish at the peak SNPs of the six detected QTLs (see Table 5 for QTL characteristics). The Y-axis represents the TLE of fish (no unit) and the three colored boxes represent the three genotypes for a given SNP (two homozygous and one heterozygous). The dots represent the fish individual phenotype.


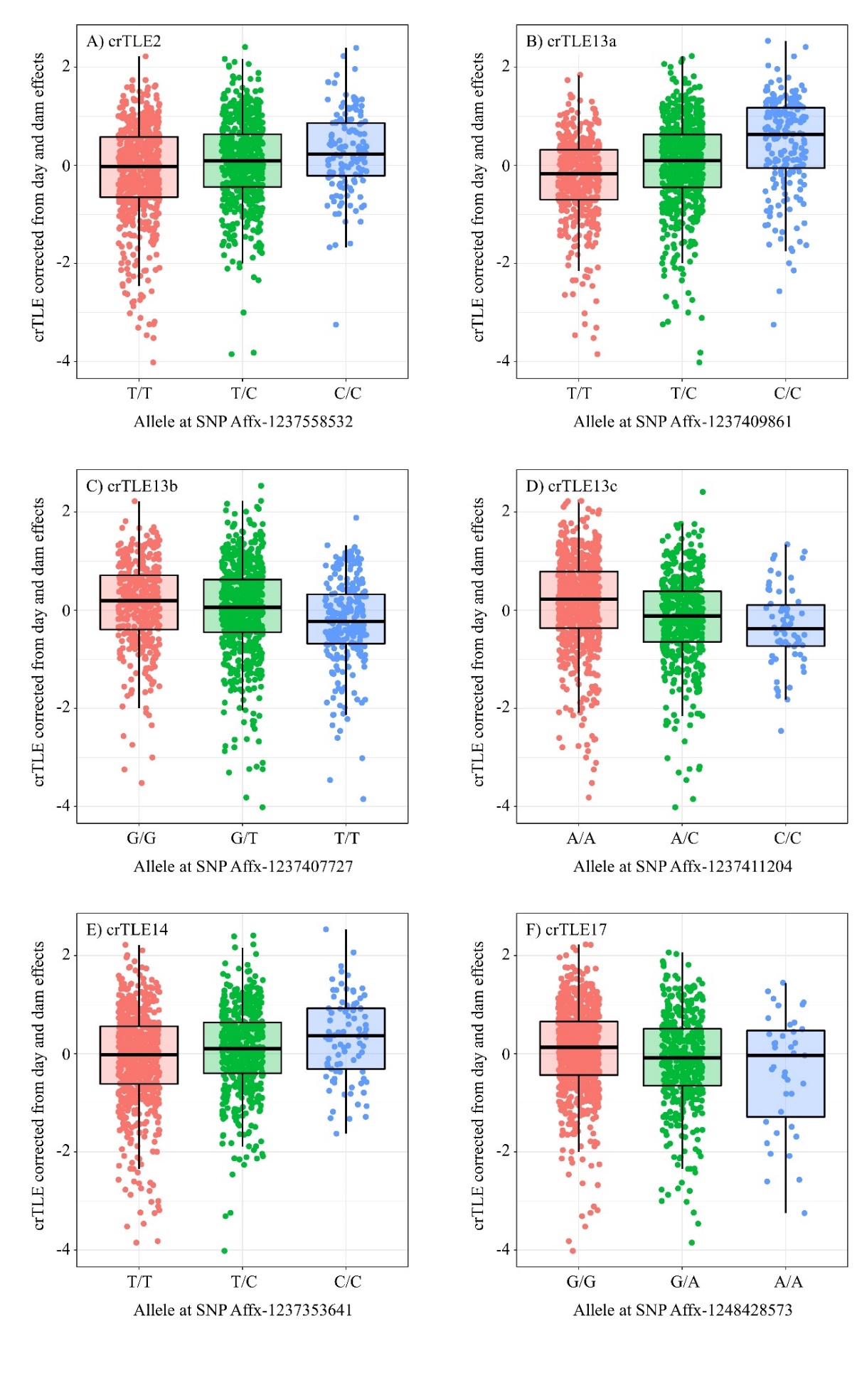


TLE17-1

TLE13-3

TLE13-1

TLE2-1

TLE13-2

TLE14-1
